# Portable Sensing Systems in Biological and Chemical Analyses: A Review of Sensor Technologies, Miniaturized Platforms, Data Processing, and Field Applications

**DOI:** 10.3390/mi17070863

**Published:** 2026-07-21

**Authors:** Hsuan-Yu Chen, Chiachung Chen

**Affiliations:** 1 Africa Industrial Research Center, National Chung Hsing University, Taichung 40227, Taiwan; wakaharu37@gmail.com; 2 Department of Bio-Industrial Mechatronics Engineering, National Chung Hsing University, Taichung 40227, Taiwan

**Keywords:** portable sensing, biosensors, chemical sensors, point-of-care testing, microfluidics, wearable sensors, field analysis, sensor validation

## Abstract

Portable sensing systems are increasingly important in biological and chemical analyses because they can provide analytical information at the point of decision-making. While traditional laboratory methods remain crucial for reference measurements, regulatory validation, and high-precision quantification, portable systems emphasize rapid response, convenience, cost-effectiveness, robustness, and relevance to decision-making. This paper views portable sensing systems as integrated analytical platforms rather than isolated sensing elements. The paper discusses recognition elements, including enzymes, antibodies, nucleic acid probes, aptamers, molecularly imprinted polymers, nanomaterials, and hybrid recognition interfaces, as well as electrochemical, optical, mass-sensitive, thermal, field-effect, and hybrid sensing technologies. Furthermore, this paper reviews platform designs, including paper-based analytical devices, chip lab systems, smartphone-assisted sensors, wearable and flexible sensors, handheld instruments, and wireless sensor networks. It explores their applications in sample handling, calibration, data processing, and field deployment. Applications of this technology include point-of-care diagnostics, pathogen detection, wearable health monitoring, agriculture, veterinary medicine, environmental monitoring, food safety, industrial process control, forensic analysis, public safety, and occupational exposure assessment. The report focuses on sample acquisition, miniaturized preparation, reagent storage, matrix interference, calibration transfer, signal conditioning, machine learning, cloud platforms, analytical validation, and decision support. Furthermore, it identifies key obstacles to translating academic prototypes into industrial products, including reproducibility, stability, manufacturability, ease of use, cybersecurity, regulatory approval, and market acceptance. Future development requires fully integrated sample-to-result systems, multimodal sensing, artificial intelligence, sustainable single-use materials, self-powered devices, and system-level validation under real-world operating conditions.

## 1. Introduction

Portable sensing systems have become an important direction in biological and chemical analyses because they bring analytical capability closer to the place where decisions must be made [1,2,3,4]. Conventional laboratory-based analysis has long depended on instruments such as chromatography systems, mass spectrometers, fluorescence spectrometers, polymerase chain reaction platforms, laboratory immunoassay analyzers, and benchtop electrochemical workstations. These instruments remain indispensable for reference measurement, regulatory confirmation, advanced research, and traceable quantification because they provide high sensitivity, selectivity, reproducibility, and analytical accuracy [1,2]. However, many practical decisions cannot wait for samples to be collected, transported, processed, analyzed, interpreted, and reported through centralized laboratory channels [2,3]. In clinical practice, a delayed result may postpone diagnosis or treatment [1,3]. In environmental monitoring, delayed detection may allow pollution to spread before intervention [2]. In food safety, waiting for laboratory confirmation may delay product release or allow contaminated products to enter the market [3]. In agriculture and animal production, late detection of nutrient deficiencies, pathogen invasions, pesticide residues, or disease outbreaks may reduce yield, product quality, and market value [1,2,3,4]. In industrial and occupational safety, delayed chemical or biological detection may expose workers to hazardous gases, toxic residues, or infectious materials [1,3]. These examples illustrate that the value of an analytical result depends not only on its accuracy but also on when and where it becomes available [1,3,4].

The central motivation of portable sensing is therefore not to replace all laboratory instrumentation, but to create analytical capability that is close enough to the problem to support timely action [1,2,3,4]. This motivation has shifted the concept of biological and chemical analyses from a laboratory-centered activity to a distributed information system in which sensing, interpretation, communication, and decision-making can occur near the point of sampling [3,4]. Portable sensing systems are not simply miniaturized versions of conventional laboratory instruments. They represent a different analytical philosophy [1,4]. The laboratory approach emphasizes controlled conditions, specialized operators, standardized protocols, and reference-grade instrumentation. The portable approach emphasizes accessibility, immediacy, simplicity, robustness, affordability, and relevance to decisions [2,3]. A portable device may not always match the analytical performance of a reference laboratory method, yet it can provide useful information within minutes at the sampling site [1,3]. In many real situations, a slightly less sensitive but timely result may be more valuable than a highly sensitive result that arrives after the decision window has closed [2,3].

The convergence of several technological fields has enabled the rapid development of portable sensing systems [5,6,7]. Advances in materials science have enabled the development of nanomaterials, conductive polymers, flexible substrates, catalytic nanostructures, and molecularly imprinted polymers that enhance recognition, signal amplification, and device flexibility [6,7,8,9]. Advances in molecular biology and biochemistry have enabled the development of enzymes, antibodies, nucleic acid probes, aptamers, CRISPR-associated recognition systems, and isothermal amplification methods for selective detection of biological and chemical targets [5,6,10,11,12]. Microfabrication and microfluidics have enabled small-volume sampling, mixing, separation, reaction, and detection within compact platforms [10,11,12]. Consumer electronics have contributed low-cost cameras, light sources, processors, batteries, wireless communication modules, and user interfaces that can be integrated into handheld, wearable, or smartphone-assisted devices [13,14,15,16,17,18]. Cloud computing and artificial intelligence have further expanded the role of portable sensors by supporting pattern recognition, calibration transfer, data fusion, remote monitoring, predictive analytics, and decision support [13,15,17]. Together, these developments have transformed portable sensing from simple qualitative test strips into increasingly integrated analytical platforms [4,11,12,19].

A system-level review of portable sensing must therefore consider more than sensor chemistry or transducer design [4,20]. Many academic studies emphasize the recognition element or sensing interface and report impressive limits of detection, linear ranges, response times, and selectivity tests [5,6,7,8]. These analytical metrics are necessary, but they do not, by themselves, determine whether a device can be used reliably by non-specialists in real-world settings [1,3,21]. A field-ready portable sensing system must also collect and prepare samples, tolerate complex matrices, maintain reagent stability, preserve calibration, operate under varying temperature and humidity conditions, resist vibration and mechanical damage, manage power consumption, communicate data securely, and present results in an interpretable form [3,4,11,12,22,23,24,25]. The performance of the complete system may differ substantially from the performance of the sensing element alone [4,26,27,28]. For this reason, successful portable sensing requires integrated engineering of recognition chemistry, sample handling, transduction, electronics, software, packaging, user interaction, validation, and workflow compatibility [4,10,11,13].

The attractive images, device photos, sensor sites, and experimental results of the portable sensing systems in biological and chemical analyses would help the review move beyond abstract sensor principles and demonstrate how portable sensing systems appear, operate, and perform in real biological, chemical, clinical, environmental, agricultural, food-safety, and industrial applications. The reviewed literature shows that attractive visual materials for a review on portable sensing systems can be organized around device photographs, sensing platforms, field-use sites, and experimental results. Wearable biosensor images are especially useful because they show flexible sweat patches, wristbands, epidermal sensors, and on-body monitoring systems that directly connect sensing technology with real users [17,18,29,30]. Battery-free skin-interfaced microfluidic/electronic devices provide particularly strong examples because they combine sweat collection, colorimetric analysis, electrochemical detection, wireless communication, and real-time physiological monitoring in a visible device format [18]. Microfluidic and paper-based analytical devices are also visually attractive because they clearly show miniaturized channels, reaction zones, capillary flow, and low-cost disposable platforms for point-of-care, environmental, and food-safety analysis [11,12,24,31,32]. Smartphone-based sensing images are valuable because they demonstrate how ordinary mobile phones can serve as optical readers, electrochemical interfaces, image processors, data storage units, and communication gateways for decentralized analysis [13,14,15,16]. CRISPR-based diagnostic figures can present sample preparation, nucleic acid amplification, guide-RNA recognition, reporter cleavage, and lateral-flow or fluorescence readout as an integrated molecular workflow [20,21,33]. Commercial point-of-care microfluidic devices and cartridge-based platforms are useful for discussing the translation of academic prototypes into real products, especially because they emphasize manufacturability, user operation, sample-to-answer integration, and regulatory requirements [4,19,34]. Images related to food safety and environmental monitoring can illustrate sensor use in practical settings such as water systems, farms, food-processing chains, and field inspection settings [22,23,24,25,26,27]. Experimental-result panels are also important because they provide quantitative evidence, including calibration curves, electrochemical current responses, fluorescence intensity changes, colorimetric gradients, recovery tests, and continuous wearable monitoring profiles [25,26,28,35,36].

This review examines portable sensing systems in biological and chemical analyses from this integrated perspective [1,4]. The discussion begins by defining the concept and scope of portable sensing systems. Then it reviews major recognition elements, signal transduction technologies, platform architectures, sample collection and preparation methods, calibration procedures, data processing, and decision-support approaches [5,6,7,8,9,10,11,12,13,14,15,16,28,35,36]. Applications are considered in point-of-care diagnosis, pathogen detection, wearable health monitoring, agriculture, animal health, environmental monitoring, food safety, industrial process control, forensic analysis, public safety, and occupational health [1,2,3,17,18,20,21,22,23,24,25,26,27]. The review also discusses analytical validation criteria, technical limitations, and the persistent gap between academic proof-of-concept devices and industrially deployable products [3,4,19]. Finally, emerging trends are examined, including artificial intelligence, multimodal sensing, digital monitoring networks, self-powered devices, sustainable disposable materials, and fully integrated sample-to-answer systems [18,19,28,35,36]. The central argument of this review is that portable sensing systems will achieve their greatest impact when they are designed not as isolated laboratory demonstrations, but as complete decision-support platforms for real-world biological and chemical analyses [3,4,19].

The main directions for development in portable sensing research for 2025–2026 are outlined. Microfluidics and electrochemical point-of-care testing platforms are considered practical approaches for detecting biomarkers in biological fluids, especially when sample processing, miniaturization, and rapid reading are integrated into a single device [31]. Digitally connected biosensing extends this concept by combining sensors with wireless communication, cloud systems, cybersecurity, and remote decision support [37]. AI-integrated optical biosensors further improve the accuracy of image analysis, signal interpretation, and point-of-care diagnostics [38]. Paper-based microfluidic devices are gaining attention for their low cost, single-use nature, and suitability for field testing in food spoilage and food safety monitoring [32]. Wearable sensors and biosensors focus on continuous health monitoring, non-invasive analysis of biological fluids, comfort, stability, and point-of-care data acquisition [29,30]. Other reviews explore translation and commercialization, particularly the large-scale production, supply chain control, regulatory quality, and market acceptance of electrochemical biosensors [34]. A recent review of infectious disease biosensors summarized rapid point-of-care testing strategies [33]. Electrochemical sensors and label-free biosensors based on molecularly imprinted polymers (MIPs) have made progress in terms of selectivity, gene sensing, multiplexing, biomedical applications, and commercial potential [39,40].

This review article should employ a descriptive, scope-defined review approach, combined with an applied systems engineering perspective. Its aim is not only to summarize biosensor research but also to integrate portable sensing systems into a unified platform from sample to decision. The search scope should cover publications from approximately 2010 to 2026, with a focus on the latest advancements in miniaturization platforms, field analysis, data processing, and validation.

Major databases may include Web of Science, Scopus, ScienceDirect, SpringerLink, PubMed, Google Scholar, IEEE Xplore, MDPI, Taylor & Francis, and SAGE Journals. For specific search strategies, please refer to the search source structure in the methodology document.

Search keywords should combine terms such as sensor, platform, sample, data, and application, for example, portable sensing, biosensors, chemical sensors, point-of-care testing, microfluidics, paper-based analytical devices, lab-on-a-chip, smartphone sensors, wearable sensors, sample preparation, calibration, signal processing, machine learning, decision support, validation, field analysis, environmental monitoring, food safety, agriculture, clinical diagnostics, and industrial monitoring.

Studies exploring portable platform design; sample acquisition and preparation; reagent storage; matrix interference; calibration transfer; data processing; analytical validation; decision support; manufacturability; field deployment; or the gap between academic prototypes and industrial products should be selected. Studies focusing solely on identifying chemistry or signal transduction without considering system-level portability or field relevance should be chosen with caution.

Unlike many existing reviews of biosensors, this review shifts its focus from single sensing components to complete portable analytical systems. Past reviews have typically focused on identification elements, such as enzymes, antibodies, nucleic acid probes, aptamers, molecularly imprinted polymers, and nanomaterials, or signal transduction methods, including electrochemical, optical, thermal, quality-sensitive, and field-effect sensors. These topics explain how targets are detected and converted into signals, but usually focus on the sensor itself.

This article adopts a broader, system-level perspective, viewing portable sensing as a platform from sample to decision. A practical portable device must not only detect analytes but also acquire and prepare samples, process reagents, adapt to complex matrices, maintain calibration, process signals, transmit data, and present results to support timely decision-making. Therefore, portability encompasses not only small size but also simplicity, robustness, workflow compatibility, field reliability, and decision relevance.

This review also emphasizes the importance of validation and translational applications. This report not only compares detection limits, sensitivity, selectivity, and reaction time, but also explores reproducibility, drift, matrix interference, calibration transfer, stability, manufacturability, cybersecurity, regulatory compliance, and market acceptance. Therefore, its unique contribution lies in its focus on practical applications. The report elucidates how portable sensors can become reliable, easy-to-use, proven, and deployable tools for biological and chemical analyses.

**Figure 1:**
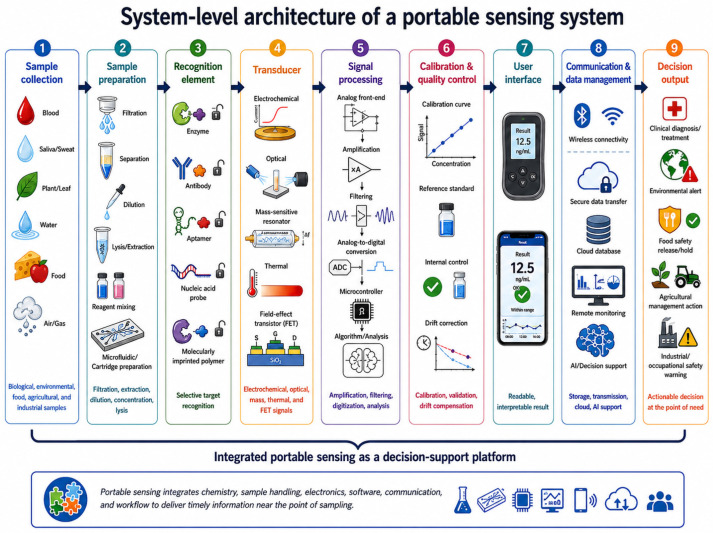
System-level architecture of a portable sensing system.

Figure 1 shows the system-level architecture of a portable sensing system, including sample collection, sample preparation, recognition element, transducer, signal processing, calibration, user interface, communication, and decision output.

## 2. Concept and Scope of Portable Sensing Systems

Portable sensing systems represent a broad family of analytical technologies that integrate chemistry, biology, materials science, electronics, mechanical design, software, data communication, and field operation [29,30,31,32,37,38]. Their central purpose is to bring biological and chemical analyses closer to where samples are generated and decisions are made [33,34,38,39,40]. The scope of portable sensing is intentionally broad because the targets, matrices, concentration ranges, users, and decision requirements differ greatly among healthcare, agriculture, food safety, environmental monitoring, industrial inspection, public safety, and personal health applications [33,34,39,40,41,42]. A disposable lateral-flow strip, a handheld electrochemical meter, a wearable sweat sensor, a smartphone-assisted optical reader, a portable spectrometer, a microfluidic pathogen-detection chip, and a wireless gas-monitoring node may appear very different in design [29,30,32,38,43,44,45,46,47,48]. However, they share the same essential function: they generate analytical information at or near the sampling site under conditions that are less controlled than those in a conventional laboratory [31,37,38,39,40].

### 2.1. Definition of Portable Sensing Systems

A portable sensing system may be defined as a compact analytical device or platform capable of detecting biological or chemical targets at or near the sampling site and converting the detection event into information that supports action [31,37,38]. This definition emphasizes that portability is not determined by physical size alone. A small instrument that still requires refrigerated reagents, complicated sample preparation, highly trained operators, controlled temperature, and expert interpretation may be transportable but not truly field-deployable [38,39,40]. Conversely, a slightly larger instrument may serve as a practical, portable sensing system if it integrates sample introduction, preparation, detection, signal processing, result interpretation, and communication into a rugged, user-friendly form [32,39,49,50]. Therefore, portability should be understood as a combination of physical mobility, analytical function, operational simplicity, environmental tolerance, workflow compatibility, and decision relevance [30,38,39,40].

The scope of portable sensing includes both biological and chemical analyses [31,37,49,50]. In biological analysis, targets may include metabolites, proteins, nucleic acids, hormones, enzymes, inflammatory markers, cells, pathogens, toxins, plant biomarkers, animal health indicators, and microbial contamination [29,34,37,38,40]. In chemical analysis, targets may include gases, ions, heavy metals, nutrients, pesticides, antibiotics, explosives, volatile organic compounds, industrial chemicals, water contaminants, food adulterants, and environmental pollutants [31,33,34,45,46,47,48]. The boundary between biological and chemical sensing is often not strict. Biological recognition elements such as enzymes, antibodies, aptamers, and nucleic acid probes may be used to detect chemical targets, while chemical transduction methods such as electrochemical, optical, thermal, and field-effect detection may be used to read biological interactions [37,49,50]. For this reason, portable sensing systems are best regarded as integrated analytical platforms rather than devices belonging to a single disciplinary category [32,39,49,50].

### 2.2. Differences Between Laboratory-Based and Portable Analysis

Portable sensing systems should be compared with laboratory-based analysis according to the decision they are intended to support, rather than by asking whether they can replace reference laboratories in all situations [38,39,40]. Centralized laboratory instruments provide excellent sensitivity, selectivity, reproducibility, calibration traceability, multiplexing capability, and data quality. They are essential for confirmatory testing, regulatory enforcement, advanced research, and complex quantitative analysis [31,37,38]. However, laboratory analysis usually requires capital-intensive equipment, trained technicians, controlled infrastructure, scheduled maintenance, sample transportation, and time-consuming pretreatment. These requirements may delay the analytical result and reduce its value in situations that require immediate action [38,39,40].

Portable sensing systems generally involve a different balance of performance and usefulness. They offer speed, accessibility, low cost per test, on-site decision support, and the ability to monitor conditions repeatedly or continuously [29,30,33,34,38]. Their limitations may include lower accuracy, narrower dynamic range, weaker matrix tolerance, device-to-device variability, reagent instability, sensor drift, limited calibration transfer, and greater sensitivity to environmental conditions [30,39,40,49,50]. A realistic evaluation should therefore ask what level of analytical performance is sufficient for the intended decision, how much uncertainty can be tolerated, whether the result should trigger immediate action or laboratory confirmation, and how the portable measurement fits into the larger workflow [38,39,40]. In many applications, a rapid on-site screening result may be more valuable than a more accurate result that arrives after the opportunity for intervention has passed [33,34,38,39,40].

Table 1 summarizes differences in accuracy, speed, infrastructure, user skill, cost, calibration, traceability, decision proximity, the limit of detection (LOD), and validation. This would support the paper’s core distinction between centralized laboratory analysis and decentralized field sensing.

### 2.3. System-Level Components

Portable sensing systems consist of several interdependent functional components [30,32,39,49,50]. The first component is a recognition element that selectively interacts with the target analyte. This element can be an enzyme, antibody, nucleic acid probe, aptamer, receptor, molecularly imprinted polymer, microorganism, cell-based recognition unit, or functional nanomaterial [37,49,50]. However, in practical applications, the recognition element is often the primary cause of performance degradation. Enzymes may lose activity during storage or under high-temperature and high-humidity conditions. Antibodies may exhibit batch-to-batch variability, cross-reactivity, or denaturation. Aptamers and nucleic acid probes may be affected by pH, ionic strength, nuclease degradation, or incomplete hybridization. While molecularly imprinted polymers are relatively stable, they may have problems such as slow binding, incomplete template removal, or cross-reactivity with structurally similar compounds. Therefore, recognition performance reported in buffer solutions may not be representative of their performance in blood, saliva, sweat, wastewater, plant juices, food extracts, or industrial liquids [29,30,33,34,39,40,49,50].

The second type is the sensor, which converts identified events into measurable signals, such as current, voltage, impedance, light intensity, color change, fluorescence, mass shift, thermal response, magnetic signals, or field-effect modulation [31,37,49,50]. In practical applications, sensors are also affected by field conditions. Electrochemical sensors may suffer from electrode contamination, redox interference, reference electrode instability, and drift. Optical sensors may be affected by ambient light, turbidity, sample color, camera variations, and optical path instability. Mass-sensitive sensors are sensitive to vibration, nonspecific adsorption, and humidity. Field-effect sensors may be affected by Debye shielding, surface instability, and inter-device variations. These limitations indicate that signal conversion must be evaluated in real-world operating environments, not just under controlled laboratory conditions [30,39,40,49,50].

The third module is the sample processing module, which may include collection, filtration, dilution, lysis, extraction, separation, mixing, pre-concentration, washing, reagent storage, or waste disposal [32,39,41]. This module is particularly important because real-world samples such as blood, sweat, saliva, soil extracts, wastewater, plant sap, food homogenization, and industrial wastewater often contain interfering substances not present in laboratory buffer tests [29,30,33,34,41,49]. Matrix interference is one of the main reasons why portable sensing systems cannot reproduce laboratory performance. Whole blood contains cells, proteins, lipids, salts, and redox-active compounds. Food samples may contain fats, pigments, fibers, proteins, and preservatives. Environmental samples may contain humic substances, suspended solids, pH variations, heavy metals, and microbial debris. Plant sap and soil extracts may contain phenols, salts, organic acids, and particulate matter. These components can hinder recognition sites, contaminate electrodes, quench fluorescence, alter viscosity, inhibit amplification reactions, or cause nonspecific signals. Therefore, sample preparation is not a secondary step; it is a key factor that determines actual accuracy, recovery, linearity, and detection limit [29,30,32,33,34,39,40,41,49,50].

The fourth component is the electronics and power module, which is responsible for acquiring, amplifying, filtering, digitizing, storing, and powering measurement data [30,47,48,49,50]. In practical applications, insufficient electronic component performance, unstable power supply, temperature sensitivity of circuits, poor shielding, or inadequate signal filtering can all increase noise and reduce the repeatability of measurement results. Wearable devices and field equipment are particularly susceptible to motion artifacts, battery aging, humidity, dust, vibration, and unstable wireless connections. These problems can lead to unstable baselines, false alarms, data loss, or shortened lifespan [29,30,47,48,49,50].

The fifth module is the computation and user interface module, which converts raw sensor signals into readable and interpretable information. This functionality may include calibration, temperature correction, signal normalization, quality control, pattern recognition, uncertainty estimation, and the display of actionable results [30,39,47,48,49,50]. In practical applications, data processing algorithms must handle drift, matrix effects, nonlinear responses, outliers, user errors, and instrument variations. Calibration models developed based on limited laboratory datasets may not generalize well across batches, devices, climates, sample types, or user groups. Machine learning can improve pattern recognition capabilities, but it can also fail when training data is narrow, biased, or unrepresentative of field samples. Therefore, decision-support outputs should include quality-control metrics, uncertainty estimates, error warnings, and recommendations for confirmatory testing when results approach critical thresholds [28,30,35,36,39,40].

The sixth module is the communication and data management module, which may include smartphone connectivity, Bluetooth or wireless transmission, cloud storage, geotagging, cybersecurity, remote monitoring, and connectivity with electronic health records, farm management systems, environmental databases, or industrial control platforms [30,43,44,48]. While connectivity enhances the value of portable sensing, it also introduces new limitations. Data may be lost due to poor network coverage, software incompatibility, battery failure, or cloud service interruptions. Privacy, cybersecurity, data ownership, and regulatory compliance become critical when transmitting clinical, professional, or industrial data. Furthermore, even if sensor results are technically correct, their practical value may be limited if decision-making information is unclear, the user interface is confusing, or the results are not integrated into actual workflows [30,37,43,44,48].

The performance of portable sensing systems depends on interactions among components, not just on the sensing material itself [30,32,39,49,50]. Highly sensitive identification layers cannot compensate for problems such as poor sampling, unstable reagents, weak packaging, unreliable calibration, inconsistent outputs, or insecure data processing [30,39,40,48,49,50]. Therefore, system-level performance should be evaluated using actual samples, recovery tests, reaction times, storage stability, inter-device repeatability, robustness to temperature and humidity variations, user-operation tests, and comparisons with accepted reference methods. Without such validation, portable sensors remain only promising prototypes, not reliable practical analytical tools [29,30,31,32,33,34,37,38,39,40].

### 2.4. Scope of Application and Proximity to Decision-Making

The applications of portable sensing technology can also be categorized by decision distance [29,30,33,34,38,39,40]. Clinicians, nurses, nursing staff, or patients can use bedside systems to assist with diagnostic, monitoring, or treatment decisions [29,38,40]. The main advantage of bedside applications is the ability to take rapid clinical action, but the risks are also higher. Interference from matrices such as whole blood, serum, saliva, urine, or sweat can affect the accuracy of test results. Improper sampling, insufficient sample volume, improper sampling time, drug interference, changes in hematocrit, and user misunderstanding can all lead to misleading results. Therefore, bedside portable sensing technology requires rigorous validation, clear operating instructions, built-in quality control, and well-defined laboratory validation rules [29,30,38,39,40].

Field testing systems are used by inspectors, farmers, veterinarians, food safety personnel, and environmental officials to assess samples directly in the field [33,34,41,42]. In these applications, field conditions are often less controlled than in clinical settings. The composition of homogenized food, soil extracts, water samples, animal body fluids, and plant materials can vary considerably. Turbidity, pigments, fiber, fat, salts, microorganisms, organic matter, and pesticide residues can interfere with measurements such as optical, electrochemical, immunological, or nucleic acid detection methods. Recovery rates may be acceptable in laboratory samples with added standards, but lower in naturally contaminated samples. Therefore, field testing techniques should use representative real samples, account for seasonal variations, be validated by different operators, and be compared with standard laboratory methods [33,34,41,42,49,50].

Operators use near-process systems to monitor industrial production lines, water treatment systems, fermentation processes, storage facilities, and greenhouse environments [33,34,45,46,47,48]. Near-process sensing emphasizes repetitive or continuous measurements, but long-term operation can introduce numerous problems, including scaling, sensor drift, biofilm formation, reagent depletion, vibration, temperature fluctuations, humidity, dust, and chemical corrosion. In industrial or agricultural environments, sensors may be exposed to cleaning agents, aerosols, fertilizers, solvents, gases, or suspended particulate matter. These factors can shorten sensor life and reduce calibration stability. Therefore, near-process systems require robust packaging, automated calibration checks, maintenance plans, drift correction, and alarm management strategies [33,34,45,46,47,48,49,50].

Proximity monitoring systems are worn or carried by individuals to monitor health status, occupational exposure, fatigue, hydration, metabolic status, or environmental risks [29,30]. Wearable personal sensors face additional limitations because the samples themselves may be unstable or indirectly related to blood concentrations. Sweat secretion rates, skin temperature, movement, evaporation, skin product contamination, biomarker transmission delays, and poor skin contact can all affect wearable biosensor readings. Mechanical deformation, biocontamination, adhesive failure, and user discomfort can also limit their long-term performance. Therefore, proximity sensing needs to be carefully interpreted, physiologically calibrated, comfort-tested, and validated in real-life activities, rather than just under controlled movement or laboratory conditions [29,30].

This classification indicates that even when using the same sensing principle, different packaging, calibration, data output, quality control, regulatory compliance, and user training may be required depending on the device user and subsequent operations [30,38,39,40]. For example, electrochemical sensors used in laboratory kits, food testing kits, greenhouse monitoring nodes, and wearable patches may be based on similar signaling principles. Still, the matrix effects, response-time requirements, stability requirements, and acceptable error levels vary by application. Therefore, the application scenario determines whether portable detection results are suitable for diagnosis, screening, early warning, process adjustment, or only for preliminary investigation [29,30,33,34,38,39,40].

Therefore, a system-level perspective extends the concept of portable sensing beyond analytical chemistry [30,32,39,49,50]. It encompasses equipment engineering, sample logistics, human factors, production repeatability, data management, regulatory compliance, and integration with operational processes [30,38,39,40,48]. Sensitivity measured in buffer solutions does not guarantee sensitivity in complex matrices [34,39,49,50]. Selectivity in testing for a few simple interfering substances does not guarantee reliability under field conditions [33,34,49,50]. A prototype operated by its inventors may not perform equally well when used by a nurse, farmer, factory worker, food inspector, or consumer [38,39,40]. This is a central limitation in the translation of portable sensing systems. Many prototypes report excellent LOD, linear range, and response time, but provide limited evidence on real-sample recovery, long-term stability, calibration transfer, batch-to-batch reproducibility, user variability, or regulatory-grade validation. As a result, their real-world performance may be weaker than their laboratory performance. The gap between academic demonstration and practical deployment can only be reduced by testing complete sample-to-answer workflows under realistic conditions [29,30,31,32,33,34,37,38,39,40].

The concept and scope of portable sensing systems must therefore be defined by their ability to deliver sufficiently accurate, timely, robust, affordable, and interpretable information at the location where that information can support a clear decision [30,38,39,40]. In practical applications, the best portable sensor is not always the one with the lowest LOD. It is the system that provides adequate sensitivity, acceptable recovery, stable calibration, manageable matrix interference, fast response, simple operation, reliable data output, and a clear decision pathway for the intended user and setting [29,30,31,32,33,34,37,38,39,40].

## 3. Recognition Elements for Biological and Chemical Detection

Recognition elements are the central components that confer selectivity on portable sensing systems. They provide the molecular, biological, or physicochemical interaction that allows a target analyte to be distinguished from other substances in real samples. In biological and chemical analyses, recognition may be based on enzymatic catalysis, antigen–antibody binding, nucleic acid hybridization, aptamer folding, receptor–ligand interaction, molecular imprinting, catalytic nanomaterials, cell-based responses, or engineered physicochemical affinity [5,6,31,37]. The choice of recognition element strongly influences sensitivity, specificity, response time, stability, cost, shelf life, manufacturability, and field suitability. It also determines requirements for sample preparation, signal amplification, reagent storage, calibration, regeneration, and disposal [3,38]. A portable sensing device can only be reliable if its recognition chemistry remains active, selective, and reproducible during storage, transport, and use under realistic environmental conditions.

### 3.1. Enzyme-Based Recognition

Enzyme-based recognition is one of the oldest and most successful strategies in portable sensing. It relies on enzymes that catalyze specific biochemical reactions, converting a target molecule or a related substrate into a measurable product. The success of the glucose meter shows how enzyme specificity, disposable strips, electrochemical transduction, compact electronics, and simple operation can be integrated into a robust portable system [51,52]. Enzymes such as glucose oxidase, lactate oxidase, cholesterol oxidase, urease, acetylcholinesterase, tyrosinase, alcohol oxidase, and peroxidase have been used to detect metabolites, nutrients, pesticides, phenolic compounds, alcohols, urea, cholesterol, and other important targets [49,50,53].

The major advantage of enzyme-based sensing is catalytic amplification, as a single enzyme molecule can convert many substrate molecules, thereby increasing signal intensity and improving sensitivity. Enzymes also provide strong molecular selectivity through their active sites. However, field deployment is limited by biological instability. Enzyme activity can be affected by temperature, pH, humidity, drying, solvents, inhibitors, oxygen availability, and long-term storage [53,54]. Immobilization is therefore critical. Crosslinking, encapsulation, adsorption on nanomaterials, covalent attachment, lyophilization, protective polymers, stabilizing additives, and mediator systems are used to improve stability [53,54]. In portable systems, enzyme performance must be tested not only in buffer but also in blood, sweat, saliva, food extracts, plant sap, soil leachate, wastewater, or industrial fluids [22,23,25].

### 3.2. Antibody- and Immunoassay-Based Recognition

Antibody-based recognition relies on specific binding between an antibody and its antigen. It is important for detecting proteins, pathogens, toxins, allergens, hormones, inflammatory biomarkers, mycotoxins, drug residues, and environmental contaminants [40,41,42,55]. Antibodies can bind large biomolecules and surface antigens with high affinity, making them useful in clinical and non-clinical applications. Lateral-flow immunoassays are the most familiar portable format because they provide rapid visual or reader-assisted detection with minimal instrumentation [55,56]. Immunosensors can also be combined with electrochemical, fluorescence, chemiluminescence, colorimetric, magnetic, surface plasmon resonance, or microfluidic platforms [25,49,57].

The strength of immunoassays lies in high selectivity and the ability to detect complex targets that are difficult to recognize by simple chemical interactions. However, antibodies may denature under heat, during drying, at extreme pH, or during poor storage. Batch variation, cross-reactivity, cost, and cold-chain dependence can limit field deployment [55,56,57]. Many immunoassays also require washing, labeling, incubation, or multi-step reagent addition, which complicates their use in portable settings. Antibody fragments, recombinant antibodies, nanobodies, engineered binding proteins, and synthetic binders are being explored to reduce cost, improve stability, and simplify manufacturing [58]. For portable applications, immunoassay design must balance specificity with reagent stability, operational simplicity, and compatibility with disposable or semi-disposable platforms [3,38,55].

### 3.3. Nucleic Acid-Based Recognition

Nucleic acid-based recognition is central to detecting pathogens, genetic markers, antimicrobial resistance genes, viral sequences, plant diseases, foodborne microorganisms, and environmental microbial contamination [20,21,40,42]. DNA and RNA probes recognize complementary sequences through base pairing, and their specificity can be adjusted by probe length, sequence design, hybridization temperature, salt concentration, and mismatch tolerance. This programmability makes nucleic acid recognition highly powerful. In portable systems, nucleic acid probes may be coupled with electrochemical electrodes, fluorescence reporters, lateral-flow strips, microfluidic chips, nanoparticles, or smartphone-assisted readers [13,14,15,16,32].

Isothermal amplification methods, including loop-mediated isothermal amplification, recombinase polymerase amplification, rolling circle amplification, and nucleic acid sequence-based amplification, reduce dependence on conventional thermal cycling and are more compatible with compact field instruments [59,60,61,62]. CRISPR-associated detection systems are also important because guide RNAs provide programmable sequence recognition, while collateral cleavage of reporter molecules generates amplified signals [20,21]. Despite strong sensitivity and specificity, system-level challenges remain. Field-ready nucleic acid sensing requires sample lysis, extraction, inhibitor removal, amplification, contamination control, temperature management, signal detection, and waste containment [19,32]. False positives due to carryover contamination and false negatives due to poor extraction or inhibition are major concerns. Successful platforms must integrate molecular recognition with reliable sample preparation and closed-cartridge operation [32,38].

In practical portable systems, DNA/RNA extraction is often the weakest link because real samples contain substances such as cells, proteins, polysaccharides, fats, humic substances, pigments, salts, plant phenols, food residues, or environmental debris, which can inhibit amplification or fluorescence detection [19,32,40,42]. Therefore, inhibitor removal, sample concentration, washing, and nucleic acid purification must be integrated into the device, rather than as separate laboratory steps [19,32]. Contamination control is equally crucial. Amplified nucleic acid products can easily contaminate work surfaces, reagent kits, pipettes, or operators’ hands, leading to false-positive results in subsequent tests [20,21,32,38]. Therefore, a closed-kit design, sealed reagent storage, unidirectional fluid flow, physical isolation between the extraction and amplification zones, and closed waste collection systems are essential for field reliability [19,32,38]. Negative controls, blank controls, extraction controls, and internal amplification controls should be provided to distinguish true test results from reagent contamination, extraction failure, or amplification inhibition [20,21,32,38]. The actual goal is not only nucleic acid identification, but also the complete integration from sample to result; that is, sample input, lysis, purification, amplification, signal reading, result interpretation, and processing are all completed in a simple, sealed, and user-safe process [19,32,38].

### 3.4. Aptamer-Based Recognition

Aptamers are short DNA or RNA sequences selected to bind proteins, small molecules, cells, toxins, metal ions, or pathogens. They are often described as synthetic alternatives to antibodies because they can provide high affinity and specificity while being chemically synthesized, easily modified, and potentially more stable during storage [63,64,65]. Aptamers can be designed so that target binding induces a conformational change that can be converted into electrochemical, fluorescent, colorimetric, field-effect, or nanoparticle-based signals [64,65].

Aptamer sensors are useful for targets that are difficult to detect with antibodies or enzymes, including some small molecules and toxins. Chemical synthesis allows more reproducible production than animal-derived antibodies, and functional groups can be introduced at defined positions for immobilization or labeling [63,64,65]. However, RNA aptamers may be degraded by nucleases, and even DNA aptamers can lose performance in complex matrices. Ionic strength, pH, temperature, nonspecific adsorption, and surface immobilization can alter folding and recognition [65]. Aptamers selected under ideal laboratory conditions may not work well in blood, food extracts, wastewater, plant sap, or industrial samples. Therefore, aptamer sensors require validation in the intended matrix and operating environment [6,65].

### 3.5. Molecularly Imprinted Polymers

Molecularly imprinted polymers are synthetic polymer matrices containing recognition cavities complementary in size, shape, and functional arrangement to a template molecule. During synthesis, monomers are polymerized around the target or a structural analog. After template removal, binding sites remain and can selectively rebind the target [9,66,67]. This approach is attractive for small molecules, pesticides, drug residues, endocrine disruptors, food contaminants, antibiotics, toxins, and heavy-metal complexes [66,67,68]. Molecularly imprinted polymers can be incorporated into electrodes, membranes, optical films, nanoparticles, microfluidic channels, or coatings [9,66].

Their advantages are chemical robustness, low cost, thermal stability, solvent tolerance, and long shelf life. These properties are valuable for systems stored and used outside controlled laboratories. Unlike enzymes or antibodies, imprinted polymers can often tolerate harsh conditions and may be manufactured at scale [66,67]. However, binding sites may be heterogeneous, template removal may be incomplete, mass transfer may be slow, and structurally similar compounds may cause cross-reactivity. In aqueous biological samples, binding may be weaker than in organic solvents [9,66]. Their practical value depends on rational template selection, reproducible synthesis, suitable surface architecture, and effective transducer integration. They are most useful when stability, cost, and small-molecule recognition are more important than extreme biological affinity [66,67,68].

### 3.6. Nanomaterial-Enhanced Recognition and Signal Amplification

Nanomaterials are widely used to enhance recognition interfaces and amplify signals. Gold nanoparticles support colorimetric changes, plasmonic enhancement, surface functionalization, and lateral-flow amplification. Carbon nanotubes and graphene improve conductivity, surface area, electron transfer, and electrode sensitivity. Quantum dots provide bright and tunable fluorescence. Magnetic nanoparticles enable separation, preconcentration, purification, and magnetic actuation. Metal–organic frameworks provide high surface area, tunable pores, catalytic activity, and adsorption capacity. Nanozymes mimic enzyme-like catalysis while often offering greater stability than natural enzymes [7,8,46,47,48,69].

Nanomaterial-enhanced sensing can improve sensitivity, lower detection limits, shorten response times, and enable new optical or electrochemical readouts [7,8,69,70,71]. However, nanomaterials introduce system-level challenges. Performance depends on particle size, morphology, aggregation, surface chemistry, purity, dispersion stability, and batch reproducibility [7,69]. Some nanomaterials may raise toxicity, environmental, or regulatory concerns, especially in disposable or wearable devices [72,73]. Scalable manufacturing and quality control are difficult because small changes in synthesis or surface modification can greatly affect the response. Therefore, nanomaterials should be engineered as reproducible and safe components of the complete platform, not merely as signal-enhancing additives [7,69,71].

### 3.7. Application-Specific Selection of Recognition Elements

The best recognition element is not always the one that gives the lowest detection limit under ideal conditions. It is the one that provides sufficient selectivity, stability, response time, cost, and usability for the intended decision [3,38]. A clinical diagnostic sensor may require high specificity because false results can affect treatment. An environmental screening sensor may prioritize low cost, rapid response, and broad coverage, with positive findings confirmed later [22,23,24]. A wearable sensor must emphasize biocompatibility, flexibility, antifouling properties, long-term stability, and comfort [17,18,29,30]. A food-safety sensor must tolerate proteins, fats, pigments, salts, residues, and heterogeneous samples [25,26,27]. Agricultural or veterinary sensors must remain rugged under variable temperature, humidity, dust, and sample composition [23,33].

Recognition chemistry must also be evaluated in terms of regeneration and disposability. Single-use systems can improve hygiene and simplify operation, but they require scalable manufacturing, stable reagents, and sustainable materials [11,12,19]. Reusable systems may reduce cost and waste, but they require reversible binding, surface cleaning, antifouling design, calibration stability, and drift correction [31,37,49]. A system-level view connects recognition chemistry with engineering, manufacturing, data processing, and human factors. Recognition design must be tested in real matrices, realistic storage conditions, and actual field environments [3,38]. Only when recognition chemistry is stable, selective, manufacturable, and workflow-compatible can portable sensing systems provide accurate information at the right location, within the required time, at acceptable cost, and in a form that supports clear decisions.

**Figure 2:**
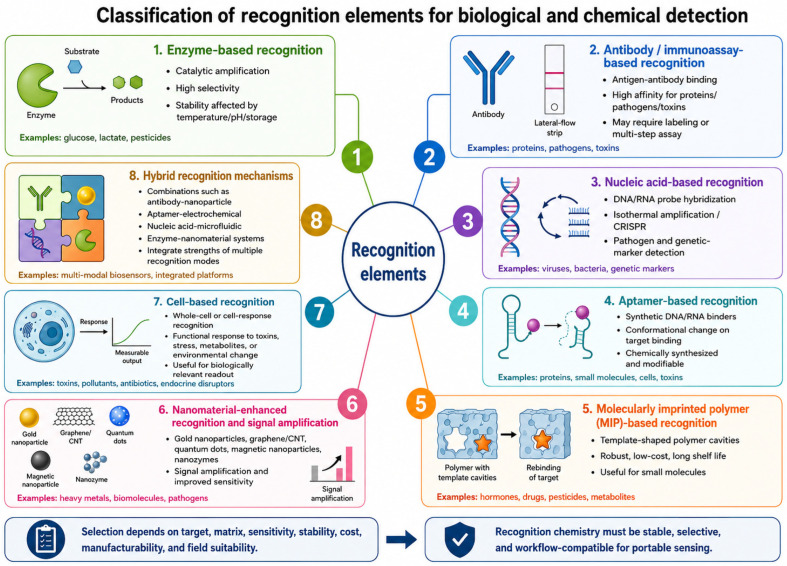
Classification of recognition elements for biological and chemical detection.

Figure 2 shows the enzyme-based, antibody-based, nucleic acid-based, aptamer-based, MIP-based, nanomaterial-enhanced, cell-based, and hybrid recognition mechanisms.

## 4. Signal Transduction Technologies

Signal transduction is the process by which a biological or chemical recognition event is converted into measurable information. In portable sensing systems, this process is not merely a measurement step but a central design decision. The selected transduction method determines device size, cost, power demand, response time, detection limit, data format, calibration requirements, user interface, and field compatibility [31,37,74,75]. Even if a recognition element provides excellent selectivity, the device will not be practical unless the transducer converts that event into a stable, interpretable, and reproducible signal under real-use conditions [3,38]. Electrochemical, optical, mass-sensitive, thermal, magnetic, and field-effect approaches each have distinct strengths and limitations. The best choice depends on the recognition element, sample matrix, required sensitivity, operating environment, user skill, and intended decision [5,6].

### 4.1. Electrochemical Sensors

Electrochemical sensing is one of the most practical and widely adopted transduction technologies for portable biological and chemical analyses. It converts chemical or biological events into measurable electrical signals, including current, potential, impedance, conductance, or charge transfer [49,50,74,75]. Amperometric sensors measure current generated by oxidation or reduction reactions and are common in enzyme-based metabolite sensors, such as glucose and lactate meters [51,52]. Potentiometric sensors measure voltage differences related to ion activity and are widely used for pH, electrolyte, and ion-selective detection [74,75].

Conductometric sensors detect changes in conductivity, while impedimetric sensors measure resistance and capacitance at electrode interfaces, making them useful for immunosensors, nucleic acid sensors, cell-based assays, and biofilm monitoring [50,76]. Voltametric methods detect current responses under controlled potential changes and are valuable for heavy metals, pesticides, redox-active compounds, and electroactive labels [22,49,75].

The strength of electrochemical sensors lies in miniaturization, low power consumption, inexpensive fabrication, and direct electronic readout [49,50]. Electrodes can be made by screen printing, thin-film deposition, carbon inks, metal patterning, or flexible electronics, enabling disposable strips, wearable patches, field kits, and handheld meters [29,77,78,79]. Signals can be acquired with compact circuits, microcontrollers, and smartphone-linked readers [13,14,15,16]. However, electrode fouling, reference electrode instability, temperature dependence, drift, nonspecific adsorption, and redox-active interferents can reduce reliability [49,50,76]. Surface modification with enzymes, antibodies, aptamers, molecularly imprinted polymers, nanomaterials, membranes, or antifouling layers can improve selectivity but may increase fabrication complexity and device variation [7,8,9,53,54]. Therefore, electrochemical portable sensors must be tested in real matrices and realistic field conditions, not only in clean buffers [22,23,25].

In practical applications, several electrochemical issues are particularly important. Electrode contamination occurs when proteins, cells, lipids, humic substances, food residues, biofilms, or reaction products adsorb onto the electrode surface, hindering electron transfer. This reduces sensitivity, alters the baseline, narrows the linear range, and increases reaction time [22,25,49,50,76]. Reference electrode instability is another limiting factor, especially in miniaturized or disposable devices. Drying, chloride-ion depletion, liquid-junction blockage, temperature variations, and matrix-dependent liquid-junction potentials can all cause shifts in the measured potential and reduce reproducibility [49,50,74,75]. Sensor drift can originate from enzymatic degradation, mediator leaching, electrode aging, surface oxidation, nanomaterial aggregation, or gradual contamination during repeated measurements [49,50,53,54,76].

Oxygen-related interference is also significant in enzyme-based amperometric sensors. In oxidase systems, oxygen acts as a natural electron acceptor; therefore, changes in dissolved oxygen can affect the signal. At negative potentials, oxygen reduction reactions generate background currents that interfere with low-concentration detection, particularly in biological fluids, wastewater, or food extracts [49,50,51,52]. Regeneration of affinity-based electrochemical sensors is challenging because antigen–antibody, aptamer–target, or molecularly imprinted polymer–analyte bindings may not be completely reversed, otherwise damaging the sensing layer [9,50,55,56,57]. Calibration transfer between different batches of sensors, instruments, temperatures, and sample matrices remains a major obstacle to field deployment [30,39,40,49,50]. Antifouling layers, selectively permeable membranes, hydrogels, nanocomposites, and protective polymers can improve stability but may reduce mass-transfer efficiency or increase variability during manufacturing [7,8,9,49,50,53,54]. Therefore, long-term stability requires a comprehensive evaluation of shelf life, operational life, reusability, storage humidity, temperature tolerance, calibration drift, and actual sample recovery [29,30,49,50,77,78,79].

### 4.2. Optical Sensors

Optical transduction converts recognition events into changes in color, absorbance, reflectance, fluorescence, chemiluminescence, phosphorescence, plasmonic response, Raman scattering, or image features [45,46,80,81]. Optical sensors are attractive for portable applications because some signals can be interpreted visually. In contrast, others can be recorded using compact readers, light-emitting diodes, photodiodes, fiber optics, or smartphone cameras [13,14,15,16,82]. Paper-based colorimetric devices, lateral-flow assays, dipsticks, and test strips are widely used because they are simple, low-cost, and require little instrumentation [11,12,55,56,57]. Smartphone-assisted optical sensing adds image acquisition, computation, storage, geotagging, communication, and user interface functions [13,14,15,16,82].

Colorimetric methods are useful when recognition produces visible changes, such as nanoparticle aggregation, enzymatic color formation, pH change, or dye displacement [7,11,12]. Fluorescence methods generally provide higher sensitivity than simple colorimetry because they can detect weak signals above background under controlled excitation [80,81,82]. Chemiluminescence and bioluminescence avoid external excitation and can achieve high sensitivity, but they require suitable reagents and timing control [80,81,83]. Surface plasmon resonance and localized plasmonic sensing can detect binding near metal surfaces [46,84,85]. Raman spectroscopy, especially surface-enhanced Raman scattering, provides molecular fingerprint information but requires reproducible substrates and compact instrumentation [45,84].

Optical sensing faces important field limitations. Visual interpretation is simple yet subjective, influenced by lighting, background color, and user perception [11,12,13]. Quantitative optical analysis must address ambient light, turbidity, scattering, background color, optical path length, camera settings, device variation, distance, angle, and reagent stability [13,14,15,16,82]. Smartphone-based assays are convenient, but variations in phone models, automatic image processing, lens properties, and illumination conditions can affect results [13,15,82]. Reliable portable optical systems therefore require internal standards, reference regions, controlled illumination, robust image processing, calibration transfer, and quality-control checks [13,14,15,16,82].

In practical applications, illumination is one of the main sources of error. Sunlight, fluorescent lights, LED spectra, shadows, and reflections all alter apparent color intensity, reducing repeatability. Smartphone cameras also vary in contrast, exposure, white balance, sensor sensitivity, and automatic image enhancement. Therefore, RGB or HSV color analysis must incorporate background correction, reference color patches, blank areas, or ratio-based signal processing to reduce variations due to device and illumination [13,14,15,16,82]. Imaging distance and angular effects are also important, as variations in camera-to-device distance, focal length, tilt angle, and optical path length all alter the captured signal. Fixed supports, enclosed imaging chambers, controlled illumination, and geometric corrections can improve reproducibility [13,14,15,16,82]. Another major limitation is interference from the true color of the sample. Blood, urine, plant sap, food extracts, wastewater, and turbid environmental samples may contain pigments, suspended particles, or colored compounds that can overlap with the analytical signal. Unless sample pretreatment, blank subtraction, spectral separation, or internal calibration is performed, these matrix colors may lead to false-positive or false-negative results [11,12,13,14,15,16,45,80,81,82].

### 4.3. Mass-Sensitive Sensors

Mass-sensitive transducers detect changes in mass, mechanical resonance, or surface stress caused by target binding. Quartz crystal microbalance devices, microcantilevers, acoustic wave sensors, and resonant microstructures are common examples [86,87,88,89]. These methods are attractive because they can provide label-free detection, allowing the binding event itself to be monitored without fluorescent, enzymatic, or electrochemical labels [86,87]. They have been applied to biomolecular interaction studies, gas sensing, pathogen detection, protein binding, environmental monitoring, and chemical vapor detection [87,88,89].

In portable sensing, mass-sensitive methods offer direct detection, but field deployment is more difficult than with many electrochemical or optical methods. Mechanical transducers often require stable oscillators, precise surface functionalization, vibration control, humidity management, and reliable fluidics [86,87,88,89]. Nonspecific adsorption can produce false signals because any added mass at the surface may affect the response [86,87]. Surface regeneration can also be difficult when strong binding or fouling occurs. Microelectromechanical systems have improved prospects for compact devices, but practical applications must still address packaging, mechanical robustness, calibration stability, and environmental compensation [87,88]. Mass-sensitive sensors are therefore most suitable when label-free detection provides a clear advantage and the operating environment can be controlled [86,87,88,89].

### 4.4. Thermal and Calorimetric Sensors

Thermal and calorimetric sensors measure heat changes associated with chemical reactions, enzyme catalysis, microbial metabolism, binding events, gas sorption, or catalytic conversion [90]. Because heat is a direct consequence of many biochemical and chemical processes, thermal transduction can provide useful information without relying on optical transparency or electroactive species [90]. Enzyme reactions, immunoreactions, microbial activity, fermentation processes, and gas sorption processes can all be monitored by measuring heat production or absorption [90]. In some cases, thermal sensing can be robust and compatible with compact devices.

The main challenge is that many recognition events produce very small heat signals. These signals can be obscured by heat loss, environmental temperature fluctuation, sensor self-heating, sample temperature differences, and poor insulation [90]. Portable thermal sensors therefore require careful thermal design, including insulation, reference channels, temperature compensation, stable baselines, low-noise temperature measurement, and controlled reaction volumes [90]. Thermal methods are often less selective unless combined with specific recognition elements such as enzymes, antibodies, receptors, or catalytic materials [5,6,90]. Their value is highest when the target reaction generates a strong thermal response or when heat measurement provides information difficult to obtain by other transduction methods [90].

### 4.5. Electrical and Field-Effect Transistor Sensors

Electrical and field-effect transistor sensors detect changes in surface charge, conductivity, gate potential, electric field, or carrier concentration caused by target binding near a sensing channel [91,92,93,94]. Ion-sensitive field-effect transistors, silicon nanowire sensors, graphene field-effect transistors, carbon nanotube transistors, organic electrochemical transistors, and chemical vapor sensors are important examples [91,92,93,94,95]. These devices are attractive because they offer label-free detection, miniaturization, direct electronic readout, and potential compatibility with semiconductor fabrication and integrated circuits [91,92,93,94]. They are promising for detecting ions, gases, proteins, nucleic acids, cells, and small molecules when suitable surface functionalization is available [92,93,94,95].

Despite this promise, field-effect sensors face major challenges. In physiological fluids and other ionic solutions, Debye screening limits the distance over which charges influence the channel, reducing signals from large biomolecules or targets away from the surface [92]. Surface functionalization must be stable, selective, and reproducible, but biological coatings may degrade or desorb during storage and field use [92,93,94]. Device variability, threshold drift, nonspecific adsorption, packaging, and fluidic integration also complicate deployment [92,93,94,95]. Wearable or implantable formats further require flexibility, biocompatibility, and long-term stability [29,30,94,95]. Field-effect sensors therefore require careful integration of surface chemistry, electronics, packaging, calibration, and matrix control [92,93,94,95].

In Table 2, the comparison includes electrochemical, optical, mass-sensitive, thermal, field-effect, magnetic, and hybrid transduction methods. Suggested columns: signal type, advantages, limitations, portability level, power demand, and common applications.

### 4.6. Hybrid and Application-Specific Transduction

No single transduction technology is universally superior. Electrochemical systems are often best for low-cost quantitative field devices because they are compact, inexpensive, and easily integrated with electronics [49,50,77,78,79]. Optical systems are attractive for visual interpretation, paper-based tests, fluorescence assays, and smartphone-assisted analysis [11,12,13,14,15,16,80,81,82]. Mass-sensitive and field-effect systems offer label-free detection but require careful engineering to overcome environmental and surface-related challenges [86,87,88,89,92,93,94,95]. Thermal systems can be direct and robust but usually need strong reaction signals and effective temperature compensation [90].

Hybrid transduction strategies are increasingly common because they combine the strengths of multiple methods. A portable assay may use magnetic nanoparticles for separation, enzymatic labels for amplification, and electrochemical readout for quantification [7,8,49,50]. Another system may combine visual colorimetric interpretation for rapid screening with smartphone imaging for digital recording and semi-quantitative analysis [13,14,15,16,82]. A wearable platform may combine electrochemical sensing with measurements of temperature, motion, and hydration to improve interpretation [17,18,29,30,95]. Hybrid systems can improve sensitivity, robustness, and decision relevance, but they also complicate calibration and validation because each component contributes uncertainty [38,95]. Therefore, transduction design in portable sensing must be guided by system-level performance rather than the isolated performance of a single signal mechanism [3,38,95].

## 5. Portable Platform Designs

Portable platform design is the stage at which sensing chemistry becomes a usable analytical product. A recognition reaction and a signal transducer may demonstrate that detection is possible, but platform design determines whether a user can obtain a reliable result outside the laboratory [3,4,19,38]. The platform defines how the sample enters the device, how reagents are stored and released, how the recognition reaction proceeds, how the signal is read, how the user interacts with the system, and how results are interpreted, stored, transmitted, or acted upon [19,96]. The same recognition chemistry may perform differently when implemented on paper, plastic microfluidics, flexible substrates, handheld instruments, smartphone accessories, or wireless sensor nodes [11,12,13,14,15,16,29,30,96]. For this reason, platform design must be considered an essential part of portable sensing rather than a secondary packaging step [4,19,38].

Portable platforms may be disposable, reusable, handheld, wearable, phone-assisted, networked, or embedded within larger monitoring infrastructures [13,14,15,16,17,18,19,29,30]. Each design reflects a different balance among cost, durability, quantitative performance, sample volume, reagent storage, power supply, calibration, data processing, user training, and maintenance [3,38,96]. A disposable test strip may be ideal for rapid screening in low-resource settings, whereas a rugged handheld spectrometer may be more suitable for inspectors who require quantitative field results [11,12,45]. A wearable sensor may emphasize comfort and continuous monitoring, whereas an Internet-of-Things sensor node may prioritize unattended operation, wireless communication, and long-term data stability [29,30]. The diversity of platform designs explains why portable sensing has expanded across healthcare, agriculture, environmental monitoring, food safety, industrial inspection, occupational safety, and personal health management [22,23,24,25,26,27,29,30,33,34].

**Figure 3:**
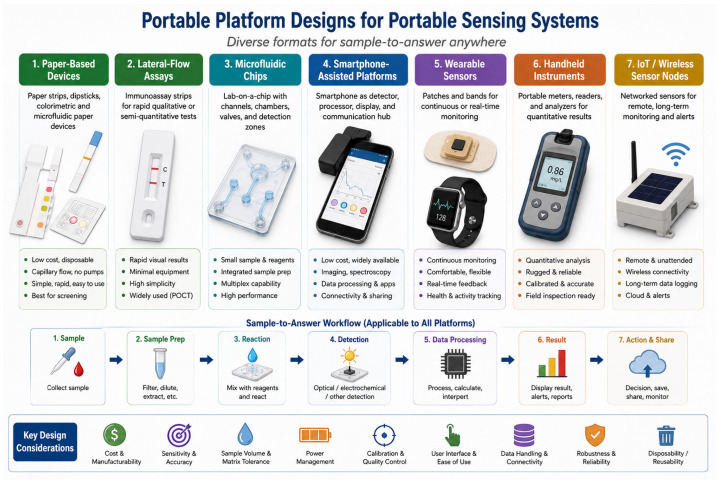
Portable platform families and representative formats.

Figure 3 shows paper strips, lateral-flow assays, microfluidic chips, smartphone readers, wearable patches, handheld meters, and IoT sensor nodes.

### 5.1. Paper-Based Analytical Devices

Paper-based analytical devices are among the simplest, lowest-cost, and most accessible portable sensing platforms. Paper and other porous substrates are lightweight, inexpensive, disposable, flexible, and naturally capable of transporting liquid by capillary action [11,12,96]. This wicking property allows fluid movement without external pumps, making paper especially useful in resource-limited settings and rapid field tests [11,12,24]. Common examples include lateral-flow assays, dipsticks, colorimetric strips, microfluidic paper-based analytical devices, and electrochemical paper sensors [11,12,55,56,57,96,97]. Patterning methods such as wax printing, inkjet printing, screen printing, laser cutting, stamping, and lamination can define channels, reaction zones, barriers, and electrode areas [96,97,98].

Paper platforms can support colorimetric, fluorescent, chemiluminescent, electrochemical, and immunoassay formats [11,12,24,96,97]. Lateral-flow immunoassays are widely known for providing rapid visual results with minimal equipment [55,56,57]. Microfluidic paper-based analytical devices can divide samples into multiple zones, enabling multiplexed detection or semi-quantitative analysis [11,12,96]. Electrochemical paper sensors can integrate printed electrodes and reagents to detect metabolites, ions, pathogens, toxins, pesticides, and environmental contaminants [22,23,24,25,26,97]. Their advantages include low material cost, ease of disposal, minimal sample volume, simple operation, and compatibility with mass production [11,12,96].

However, paper-based platforms also have limitations. Fluid flow is influenced by paper type, fiber structure, humidity, temperature, viscosity, and evaporation [96,97,98]. Complex sample preparation, washing, separation, and reagent timing are more difficult than in controlled microfluidic systems [96,97]. Reagent storage on paper can be affected by moisture, oxidation, light exposure, and temperature [96]. Quantification may be limited by uneven color development, background staining, variable lighting, and subjective visual interpretation [13,14,15,16,82,96]. Smartphone imaging or portable readers can improve quantification, but they require calibration, controlled illumination, and image-processing algorithms [13,14,15,16,82]. Therefore, paper-based devices are most suitable for applications that prioritize low cost, simplicity, disposability, and rapid screening. Still, they require careful validation when used for quantitative decision-making in complex matrices [3,38,96].

### 5.2. Lab-on-a-Chip and Microfluidic Devices

Lab-on-a-chip and microfluidic platforms manipulate small volumes of fluid through miniaturized channels, chambers, valves, pumps, droplets, membranes, mixers, separators, and detection zones [10,32,99,100,101,102]. Their purpose is to integrate sample preparation and detection into a compact device capable of performing analytical steps traditionally carried out in a laboratory [4,19,32]. Microfluidics can reduce reagent consumption, shorten diffusion distances, improve reaction kinetics, increase surface-to-volume ratio, and enable multiplexed analysis [10,99,100,101,102]. These advantages make microfluidic platforms especially valuable for nucleic acid testing, immunoassays, cell analysis, pathogen detection, wearable sweat collection, environmental preconcentration, and sample cleanup before detection [19,32,33,99,100,101,102].

Materials used in microfluidic devices include glass, silicon, polydimethylsiloxane, thermoplastics, paper, hydrogels, elastomers, and hybrid laminates [10,99,100,101,102]. Each material has different advantages and limitations. Glass and silicon provide precise structures and chemical stability but may be costly [10,102].

Polydimethylsiloxane is useful for prototyping but may absorb small molecules and is not always ideal for mass production [99,100,101]. Thermoplastics are attractive for injection molding and other commercial manufacturing processes [4,19,101]. Paper and hybrid materials provide low-cost fluid transport but less precise control [11,12,96]. The choice of material must match the sample type, reagent chemistry, fabrication scale, cost target, and intended operating environment [4,19,101].

Microfluidic devices can greatly improve portable sensing, but they also introduce practical barriers. Clogging can occur when real samples contain cells, soil particles, food residues, fibers, precipitates, or biofilms [19,32]. Bubbles may interrupt flow, alter reaction volumes, or disturb optical and electrochemical detection [19,32,101]. Evaporation is a concern in small channels. Sealing, leakage, reagent storage, sample loading, and waste containment can determine whether the device remains usable outside the laboratory [4,19,32]. A chip that works well when operated by trained researchers with syringes, pumps, pipettes, and microscopes may fail when used by a nurse, farmer, food inspector, or field technician [4,19]. Therefore, successful lab-on-a-chip design must include simple actuation, robust sample introduction, integrated reagents, clear user steps, and tolerance to real-world sample variability [4,19,32].

### 5.3. Smartphone-Based Sensing Systems

Smartphone-based sensing systems have developed rapidly because smartphones provide cameras, light sources, processors, storage, wireless communication, global positioning, touch screens, and user interfaces in a widely available device [13,14,15,16,82]. In portable sensing, smartphones can act as optical readers, electrochemical interfaces, data processors, displays, communication gateways, and cloud-connected decision tools [13,14,15,16,82]. They can read colorimetric strips, lateral-flow assays, fluorescence signals, microfluidic devices, portable microscopes, spectrometers, and phone-connected electrochemical modules [13,14,15,16,43,82,83]. They can also guide users through testing procedures, record time and location metadata, upload results, support remote consultation, and connect measurements to cloud-based databases or decision-support systems [103].

The main advantage of smartphone-based sensing is accessibility. Because many users already own smartphones, sensing systems can reduce the need for dedicated readers and make field testing more scalable [13,14,15,16,82]. Image-based colorimetry can transform visual tests into semi-quantitative or quantitative assays [13,14,15,16,82]. Smartphone fluorescence readers can use compact attachments for excitation and emission filtering [13,16,83]. Phone-connected electrochemical modules can perform measurements while using the phone for data processing and display [15,16].

However, smartphone sensing also faces serious challenges, especially for regulated or high-stakes applications. Phone models differ in camera sensors, lenses, automatic exposure control, color processing, flash intensity, operating systems, accessory dimensions, battery behavior, and software updates [13,14,15,16,82]. Ambient lighting, imaging distance, angle, focus, and user handling can affect optical measurements [13,14,15,16,82]. Apps require maintenance, cybersecurity, privacy protection, version control, and validation [19]. A smartphone may be convenient for a research prototype, but a commercial medical, food-safety, or environmental product must control variation across devices and software environments [19]. Reliable smartphone-based sensing therefore requires calibration standards, reference regions, controlled accessories, robust algorithms, secure data handling, and clear user-interface design [13,14,15,16,82].

In smartphone-based optical sensing, illumination effects are particularly important because the acquired signal depends not only on the detected color but also on external light intensity, spectrum, shadows, reflections, and flash behavior. Even if the test strip or microfluidic device remains constant, camera contrast, auto-exposure, white balance, saturation correction, and built-in image enhancement can alter the apparent signal [13,14,15,16,82]. Therefore, RGB and HSV analyses should be supplemented with background correction, blank-area subtraction, reference color patches, and ratio-based calibration. Imaging distance and angle effects are also crucial. Variations in camera-to-sample distance, focal length, tilt angle, and field of view can all alter color intensity, fluorescence brightness, and the interpretation of detection lines. Fixed supports, enclosed imaging boxes, normalized illumination, and geometric corrections can reduce these errors [13,14,15,16,82]. The true color of the sample is another practical limiting factor. Blood, urine, saliva, plant sap, food extracts, wastewater, and turbid environmental samples may contain pigments or suspended particles that can overlap with the detected color. These matrix effects require preprocessing, internal standards, spectral separation, or algorithmic correction to achieve reliable quantitative smartphone sensing [11,12,13,14,15,16,43,82,83].

### 5.4. Wearable and Flexible Sensors

Wearable and flexible sensors form a distinct class of portable platforms designed for continuous or repeated monitoring on the body, clothing, protective equipment, animals, plants, or flexible surfaces [17,18,29,30,104,105,106,107]. In human health applications, wearable biosensors may analyze sweat, interstitial fluid, saliva, breath, tears, or compounds emitted from the skin [17,18,29,30,104,105,106,107]. They may monitor electrolytes, glucose, lactate, cortisol, alcohol, hydration status, fatigue, inflammation, or exposure to toxic substances [17,18,29,30,104,105,106,107]. In occupational and environmental settings, wearable chemical sensors may monitor gases, solvents, pesticides, heat stress, humidity, strain, or personal exposure [29,30,106,107]. Flexible sensors can also be attached to plants, fruits, packages, pipelines, or industrial surfaces for localized monitoring [29,30,106,107].

The major advantage of wearable and flexible sensing is proximity over time. Instead of providing a single measurement, these systems can generate continuous or repeated data that reveal trends, variability, and exposure history [17,18,104,105,106,107]. Flexible substrates, stretchable electrodes, textile integration, soft hydrogels, printed electronics, and wireless modules have expanded the design possibilities [29,30,104,105,106,107]. Wearables can also combine biochemical sensing with physical measurements such as temperature, motion, heart rate, respiration, and skin conductance to improve interpretation [17,18,104,105,106,107].

Their limitations are substantial. Mechanical deformation, bending, stretching, motion artifacts, sweat-rate variability, skin irritation, biocompatibility, adhesive failure, biofouling, calibration drift, power supply, and wireless reliability can affect data quality [29,30,105,106,107]. Interpretation is also complex because analyte concentrations in sweat, saliva, breath, or interstitial fluid may not directly represent blood concentrations [29,30,105,107]. Physiological context, sampling rate, skin condition, local evaporation, contamination, and individual variation must be considered [105,106,107]. For this reason, wearable biochemical sensors require careful validation against reference measurements, long-term stability testing, and user-centered design that considers comfort, hygiene, data privacy, and behavioral acceptance [29,30,105,106,107].

### 5.5. Handheld and Field-Deployable Instruments

Handheld and field-deployable instruments occupy the middle ground between simple disposable tests and full laboratory equipment. Examples include portable electrochemical analyzers, gas detectors, Raman spectrometers, near-infrared spectrometers, fluorescence readers, ATP meters, lateral-flow readers, compact immunoassay readers, and portable PCR or isothermal amplification devices [19,45,74,75,108,109]. These instruments are often used by environmental agencies, food inspectors, agricultural consultants, veterinary personnel, hospital staff, industrial safety teams, emergency responders, and process operators who require more reliable or quantitative results than visual strips can provide [22,23,24,25,26,27,45,108,109].

The strength of handheld instruments is that they offer greater control over measurement conditions. A dedicated reader can manage illumination, temperature, reaction timing, optical alignment, electrical acquisition, calibration, data storage, and quality checks [45,74,75,108,109]. Rugged housing can protect sensitive components from dust, water, vibration, impact, and field handling. Battery-powered operation allows testing in locations without stable power [19,45,108,109]. Data traceability, user login, sample identification, barcode scanning, and electronic records can support regulatory or industrial workflows [19].

However, handheld instruments are more expensive than disposable platforms and require maintenance, calibration, training, software updates, and sometimes consumables [19,45,108,109]. Their practical success depends on more than analytical sensitivity. Robust housing, clear interfaces, battery life, calibration routines, data integrity, cleaning procedures, and serviceability are equally important [19]. In field use, users need results that are understandable and actionable, not merely numerical outputs [19,110,111]. Therefore, handheld instruments should be designed around the workflow of the intended user, whether that user is a greenhouse manager, wastewater operator, factory technician, clinician, food inspector, or emergency responder [19].

### 5.6. Wireless Sensor Networks and Internet-of-Things Platforms

Wireless sensor networks and Internet of Things platforms extend portable sensing from isolated measurements to distributed monitoring systems [110,111,112,113,114,115]. Instead of a single device producing a single result, multiple sensor nodes can collect data across space and time, transmit data to gateways or cloud databases, and support dashboards, alarms, maps, automated control, or predictive analytics [110,111,112,113,114,115]. Such systems can monitor rivers, farms, greenhouses, livestock facilities, cold chains, factories, hospitals, urban environments, or occupational exposure zones [110,111,112,113,114,115]. In industrial and agricultural settings, networked sensors can also be linked to control systems that adjust ventilation, irrigation, disinfection, cooling, alarms, or process parameters [110,112,115].

The value of networked portable sensing lies in temporal and spatial information. A single measurement may indicate the condition at one point, but a sensor network can reveal trends, gradients, hotspots, periodic changes, and abnormal events [110,111,112,113,114,115]. Geotagged data, cloud storage, remote access, and automated alerts can transform sensing into a decision-support infrastructure [110,111,112,113,114,115]. Wearable sensors can transmit personal exposure or physiological data [17,18,29,30,104,105,106,107]. Environmental nodes can track water quality, air pollutants, soil nutrients, or greenhouse gases [22,23,24,110,111,112,113,114,115]. Industrial sensors can feed real-time dashboards for safety and process control [110,115].

Nevertheless, connectivity introduces additional challenges. Sensor drift, calibration mismatch, data gaps, communication failure, battery depletion, environmental damage, and maintenance burden can weaken network reliability [110,111,112,113,114,115]. Cybersecurity, data ownership, privacy, interoperability, and cloud dependence must be addressed, especially when measurements influence health, safety, regulatory, or industrial decisions [110,111,112,113,114,115,116,117]. A network is only as useful as its weakest nodes, calibration strategy, data-quality controls, and decision rules [110,111,112,113,114,115]. Therefore, Internet-of-Things sensing platforms require not only good sensors but also maintenance planning, data validation, secure communication, edge computing, user dashboards, and integration with operational workflows [110,111,112,113,114,115,116,117,118].

### 5.7. Workflow, Packaging, and User-Centered Platform Design

Platform design should be guided by the intended workflow from the earliest stage of development [116,117,118]. A farmer, nurse, food inspector, wastewater operator, greenhouse manager, factory technician, emergency responder, and consumer have different time constraints, training levels, safety requirements, sample types, decision thresholds, and tolerance for uncertainty [3,38,116,117,118]. A platform designed without understanding the user’s workflow may be scientifically impressive but practically unused [116,117]. User-centered design should therefore begin before the chemistry and hardware are finalized, not after a prototype has already been built [117,118].

Packaging is an underappreciated but decisive part of platform design. The package must protect reagents, guide the user, control fluid flow, preserve alignment between sensor and reader, prevent contamination, contain waste, support storage, and withstand transportation [19,38,116,117]. In commercial portable sensing products, packaging often determines reliability as much as the sensing material itself [19,38]. Good packaging transforms fragile chemistry into an operable tool.

Poor packaging can cause leakage, reagent degradation, user error, contamination, calibration failure, or mechanical damage [19,38,116,117]. Therefore, portable platform design must integrate materials, mechanics, electronics, software, sample handling, packaging, and human factors into a coherent analytical system [19,38,116,117,118,119,120]. The ultimate goal is not only to miniaturize analysis but also to deliver sufficiently accurate, timely, affordable, robust, and interpretable information in a setting where that information can support a clear decision [3,38,116,117,118,121,122].

In Table 3, the comparison of Portable platform designs and their operational characteristics includes paper-based devices, lab-on-a-chip systems, smartphone-assisted sensors, wearables, handheld instruments, and wireless sensor networks.

## 6. Sample Collection and Preparation for Portable Analysis

Sample collection and preparation are often the determining factors in the performance of portable sensing systems. Although many academic studies emphasize the sensing surface, recognition chemistry, and analytical signal, real samples are rarely clean, uniform, or directly compatible with the sensing element [123,124]. Biological samples may contain cells, proteins, salts, enzymes, clotting factors, pigments, metabolites, and microorganisms [125,126,127]. Environmental and food samples may contain particles, oils, fats, pigments, humic substances, organic matter, preservatives, surfactants, and variable pH [22,23,24,25,26,27]. Industrial samples may contain solvents, corrosive compounds, high ionic strength, emulsions, residues, or unknown interferents. If these matrices are not managed properly, even a highly sensitive and selective sensor may produce unreliable results [123]. Therefore, portable analysis must be designed around the complete path from sampling to answer, not only around the final detection step [19,38,116,123].

The term sample-to-answer describes the ideal portable analytical workflow in which a user introduces a raw or minimally treated sample and receives an interpretable result without complex manual handling [19,124,128]. This goal is difficult because sampling error, matrix interference, reagent instability, and user variation can be as important as sensor performance [117,123]. A portable system that performs well in buffer solution may fail in blood, sweat, wastewater, soil extract, food homogenate, plant sap, or industrial effluent [22,23,24,25,26,27,29,30,123]. For this reason, sample collection and preparation should not be added as a secondary step after sensor development. They must be co-designed with the recognition element, transduction method, platform architecture, packaging, calibration strategy, and intended user workflow [19,38,116,117,123].

**Figure 4:**
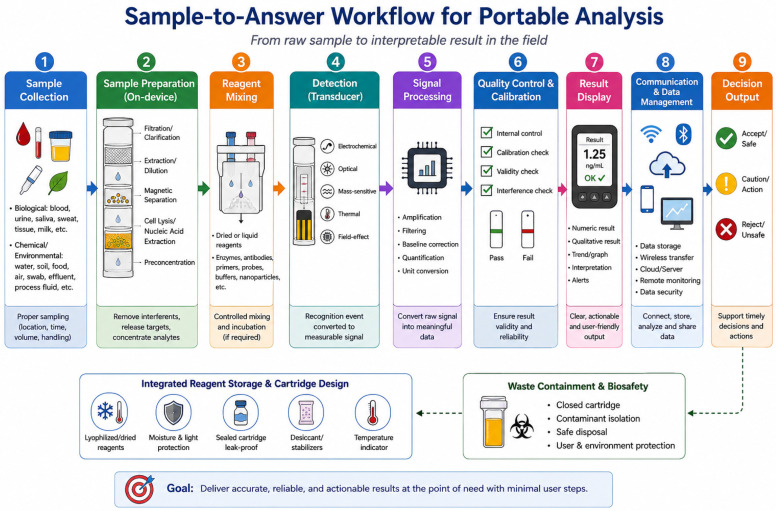
Sample-to-answer workflow for portable analysis.

The process diagram in Figure 4 shows the workflow from raw sample to interpretable result: sampling, filtration/extraction/lysis, reagent mixing, detection, signal processing, quality control, result display, and waste containment. This is highly aligned with the manuscript’s emphasis on workflow integration.

### 6.1. Biological Samples

Biological samples used in portable analysis include blood, serum, plasma, urine, saliva, sweat, tears, breath condensate, interstitial fluid, wound fluid, plant sap, tissue extracts, microbial cultures, milk, and animal body fluids [29,30,125,126,127]. Each matrix presents distinct analytical challenges [125,126,127]. Whole blood contains red and white blood cells, platelets, proteins, clotting factors, salts, lipids, and redox-active species that may interfere with electrochemical, optical, or immunological measurements [128,129,130,131]. Serum and plasma are easier to analyze than whole blood but require separation, which may be difficult in low-resource or field settings [123,127]. Urine is relatively easy to collect, but its ionic strength, pH, dilution, and metabolite composition vary widely among individuals and sampling times. Saliva contains enzymes, mucus, food residues, microorganisms, and variable viscosity [127]. Sweat is attractive for wearable sensing, but sweat rate, evaporation, skin contamination, temperature, and local physiology strongly influence measured concentrations [104,105,106,107,132,133,134].

Plant, animal, and microbial samples introduce additional complexity. Plant sap may contain pigments, phenolic compounds, sugars, organic acids, fibers, and particulates that can foul electrodes, absorb light, or interfere with recognition chemistry [22,23]. Animal fluids may contain proteins, fats, salts, pathogens, or residues of veterinary drugs [25,26,27]. Microbial samples often require concentration, washing, and lysis before nucleic acids, proteins, metabolites, or toxins can be detected [125,126,127,128]. Wound fluid and tissue extracts contain proteins, enzymes, inflammatory compounds, cellular debris, and microorganisms that may vary markedly over time and across locations [127]. These examples show that portable biological analysis requires matrix-specific preparation rather than a universal sample-handling approach [123,127,128].

### 6.2. Chemical and Environmental Samples

Chemical and environmental samples include drinking water, wastewater, seawater, river water, soil extracts, air, aerosols, food products, agricultural inputs, pesticide formulations, industrial effluents, process fluids, workplace gases, surface swabs, and residues from equipment or packaging [22,23,24,25,26,27,135,136,137,138,139]. Water samples may contain suspended solids, organic matter, microorganisms, competing ions, chlorine, metals, and variable salinity [22,23,24,137]. Wastewater is particularly challenging because it contains complex mixtures of organic compounds, detergents, microorganisms, particles, and fluctuating chemical conditions [22,23,24]. Soil extracts vary in pH, clay content, salinity, organic matter, moisture, and extraction efficiency [137,138,139]. Air monitoring requires reliable sampling of gases, vapors, aerosols, or particles, often under changing humidity, temperature, and flow conditions [137].

Food samples are among the most challenging matrices for portable sensing because they may contain fats, proteins, carbohydrates, pigments, spices, preservatives, salts, fibers, and processing residues [25,26,27,135,136]. Milk, meat, fruit juice, grains, vegetables, oils, and processed foods each require different extraction or clarification strategies [25,26,27,135]. Industrial samples may contain solvents, surfactants, oils, corrosive chemicals, high ionic strength, or suspended particles that damage sensing surfaces or distort signals [22,23,24,138]. For these reasons, portable chemical and environmental analysis often requires simplified filtration, extraction, dilution, preservation, or preconcentration before detection [24,135,136,137,138,139]. The preparation method must be simple enough for field use but strong enough to reduce matrix effects to an acceptable level [123,135,138,139].

### 6.3. Miniaturized Sample Preparation

Miniaturized sample preparation includes on-device or near-device procedures that make real samples suitable for reliable sensing. Common strategies include membrane filtration, paper filtration, sedimentation, dilution, reagent mixing, extraction, solid-phase extraction, liquid–liquid extraction, magnetic separation, electrophoresis, isotachophoresis, dialysis, immunocapture, cell lysis, nucleic acid extraction, enzymatic pretreatment, and preconcentration [123,125,126,127,128,129,130,131]. These procedures may remove interferents, release targets, concentrate analytes, adjust pH, reduce viscosity, separate cells, or deliver reagents in a controlled sequence [123,125,126,127,128].

Magnetic nanoparticles are useful in portable preparation because they can capture targets from a larger sample volume and then be concentrated into a small detection zone using a simple magnet [129,130,131]. Microfluidic channels can integrate filtration, mixing, washing, reaction, amplification, and detection, although clogging, bubble formation, sample loading, and external actuation remain concerns [99,100,101,102,103,123,128]. Paper-based devices can provide passive filtration and capillary transport, but complex multistep preparation is difficult to control [96,97,98,128]. Cartridge-based platforms can enclose reagents, guide sample flow, protect users from contamination, and contain waste [19,38,124,128]. Closed cartridges are especially important for pathogen testing and nucleic acid amplification because they reduce the risk of contamination and improve biosafety [19,32,124,128].

### 6.4. Reagent Storage and Field Stability

Reagent storage is a critical part of sample preparation in portable analysis. Many sensing systems require enzymes, antibodies, primers, nucleotides, buffers, redox mediators, fluorescent probes, nanoparticles, stabilizers, wash solutions, or extraction reagents [5,6,7,8,9,20,21,53,54]. Liquid reagents can simplify reaction chemistry but create problems related to leakage, evaporation, freezing, microbial contamination, and short shelf life [19,38,140,141]. Dried reagents improve storage and transport but must rehydrate rapidly and reproducibly [140,141,142,143]. Lyophilized reagents can preserve biological activity, but packaging must protect them from moisture, heat, oxygen, and mechanical damage [140,141,142,143]. In hot and humid environments, reagent stability may be more difficult to achieve than analytical sensitivity [3,38,140,141,142,143]. Desiccants, foil pouches, sealed cartridges, stabilizing sugars, polymers, protein protectants, and temperature indicators can help maintain performance, but they add cost and design complexity [19,38,140,141,142,143].

In Table 4, the biological, environmental, food, agricultural, industrial, and wearable samples are listed and discussed, along with their major interferents and suitable preparation methods such as filtration, dilution, extraction, magnetic separation, lysis, or preconcentration.

### 6.5. Challenges of Complex Matrices

Complex matrices interfere with portable sensing through chemical reactions, nonspecific adsorption, optical background, electrode fouling, viscosity effects, variable flow, competitive binding, pH variation, ionic strength changes, particle blockage, and biological degradation [22,23,24,25,26,27,49,50,76,123]. Optical sensors may be affected by turbidity, color, scattering, or fluorescence background [13,14,15,16,80,81,82]. Electrochemical sensors may be affected by redox-active interferents, electrode fouling, reference electrode drift, or changes in conductivity [49,50,76]. Immunoassays and nucleic acid tests may be affected by inhibitors, nonspecific binding, or poor target extraction [20,21,55,56,57,125,126,127,128]. Wearable sensors may be affected by sweat rate, skin contamination, motion artifacts, and biofouling [104,105,106,107,132,133,134].

Strategies to reduce matrix interference include sample dilution, selective membranes, antifouling coatings, filtration, washing steps, internal standards, reference channels, ratiometric signals, matrix-matched calibration, selective extraction, preconcentration, and computational compensation [24,76,123,135,136,137,138,139]. Machine-learning approaches may help correct complex interference patterns, but they require robust training data from real samples and cannot substitute for poor sample handling [28,35,36]. Each additional preparation step may improve analytical reliability but also increase user burden and the risk of error [117,123]. A practical, portable device must therefore use the simplest sample-preparation method that provides sufficient reliability for the intended decision [3,38,116,117].

Sampling error itself can be larger than sensor error [137,138,139]. A water sensor cannot represent a river if the sampling location, depth, or timing is inappropriate [137,138,139]. A plant sap test cannot represent crop status if leaves are selected inconsistently. A food residue test cannot be representative of a batch if the sample is not properly homogenized [135,136,139]. A sweat sensor cannot represent systemic physiology if sweat rate and contamination are ignored [104,105,106,107,132,133,134]. Portable sensing protocols should therefore specify sampling method, timing, location, volume, storage conditions, acceptance criteria, and quality-control checks [137,138,139]. The most successful portable sensing systems are those in which sampling, preparation, sensing, interpretation, and action are integrated into a coherent and realistic workflow [19,38,116,117,123].

## 7. Data Processing, Calibration, and Decision Support

Portable sensing systems increasingly depend on data processing, calibration, and decision-support functions as much as on recognition chemistry and hardware design. Raw sensor outputs are rarely sufficient for practical decision-making because they may contain noise, drift, baseline variation, environmental effects, matrix interference, device-to-device differences, reagent aging, and user-induced variability [144,145,146,147]. A current, voltage, color intensity, fluorescence image, impedance spectrum, mass shift, or field-effect response must be transformed into information that a user can understand and act upon [31,37,49,50]. This transformation may produce a concentration estimate, a positive or negative classification, a risk level, an alarm, a quality-control flag, or a recommended action [3,38,116]. As portable systems become connected, intelligent, and networked, data processing also supports remote monitoring, geotagging, cloud storage, longitudinal analysis, calibration management, quality control, and integration with clinical, agricultural, environmental, food-safety, or industrial decision systems [110,111,112,113,114,115,116,117]. The analytical value of a portable sensing device therefore depends on the trustworthiness of both the physical measurement and the digital interpretation [3,38].

### 7.1. Signal Conditioning and Noise Reduction

Signal conditioning is the first step in transforming raw sensor output into stable analytical information. In portable environments, signals are affected by vibration, temperature change, humidity, ambient light, electromagnetic interference, motion artifacts, sample heterogeneity, and inconsistent user handling [144,145,146,147]. Hardware-level conditioning may include amplification, current-to-voltage conversion, shielding, grounding, analog filtering, reference measurement, and temperature monitoring [144,145]. Software-level conditioning may include smoothing, baseline correction, normalization, background subtraction, outlier detection, artifact rejection, drift compensation, and feature extraction [146,147]. These procedures are essential because a sensing reaction may be chemically sound yet still yield unstable or misleading results if the signaling pathway is poorly controlled [49,50,76].

Different transduction methods require different signal-processing strategies. Electrochemical sensors may require reference electrode monitoring, baseline subtraction, compensation for temperature and ionic strength, and correction for electrode drift or fouling [49,50,76]. Optical sensors may require illumination control, white-balance correction, region-of-interest selection, background subtraction, color-space transformation, and correction for sample turbidity or uneven lighting [13,14,15,16,80,81,82]. Wearable sensors may require motion artifact removal, sweat-rate normalization, time alignment, and detection of poor skin contact [29,30,104,105,106,107]. Gas sensors may require humidity and temperature compensation because environmental changes can strongly affect adsorption, diffusion, and conductivity [47]. In all cases, signal conditioning must be evaluated under realistic field conditions, not only under controlled laboratory conditions [3,38,123].

### 7.2. Calibration and Quantification

Calibration is central to quantitative portable sensing because it relates sensor response to analyte concentration under defined conditions [148,149,150,151,152,153]. A calibration curve may be linear or nonlinear and may be based on single-point, two-point, or multi-point calibration [148,149,150]. Some applications require full quantitative analysis, while others require only a threshold decision or semi-quantitative category [3,38,116]. For portable systems, calibration is difficult because the relationship between signal and concentration may change with device batch, reagent age, temperature, humidity, sample matrix, user operation, and storage history [123,151,152,153]. A calibration curve developed with one prototype in a laboratory may not be valid for another device produced in a different batch or used in a different environment [151,152,153].

Several strategies can improve calibration reliability. Internal standards can correct for variations in sample volume, optical intensity, reagent activity, or signal recovery [148,149,150,151,152,153]. Reference electrodes, reference colors, reference channels, and ratiometric optical signals can reduce environmental and device variation [13,14,15,16,49,50,80,81,82]. Standard addition can improve quantification in complex matrices, although it may be too complicated for many field users [148,149]. Onboard calibration materials and self-check algorithms can identify device failure or reagent degradation before results are reported [116,117]. Calibration transfer is especially important for commercial and networked systems because many devices may be manufactured at different times, distributed to different locations, and used by different operators [151,152,153]. Without reliable calibration transfer and drift correction, large-scale deployment can produce data that appear precise but are not comparable [151,152,153].

### 7.3. Machine Learning and Pattern Recognition

Machine learning and pattern recognition are increasingly used to interpret complex, multivariate, or drifting sensor outputs [28,35,36,154,155,156,157,158,159,160,161]. Classification algorithms can distinguish positive and negative results in lateral-flow images, fluorescence patterns, electrochemical profiles, spectral signals, sensor arrays, or electronic noses [13,14,15,16,28,35,36,159]. Regression models can estimate analyte concentrations from multivariate data [148,149,150,151,152,153]. Anomaly detection methods can identify sensor failures, unusual samples, invalid tests, or out-of-range conditions [154,155,156,157,158]. Sensor-fusion approaches can combine electrochemical, optical, temperature, humidity, flow, motion, location, and contextual data to improve reliability [28,30,35,36,95]. Domain adaptation and transfer learning may facilitate calibration transfer across devices, batches, users, or environmental conditions [151,152,153,160,161].

However, machine learning does not eliminate the need for robust chemistry, good sampling, reliable calibration, and transparent validation [28,35,36,123]. A model trained on narrow laboratory data may fail when exposed to real-world matrices, temperature variation, user errors, degraded reagents, or unexpected interferents [123,154,155,156,157,158]. Overfitting is a major risk when datasets are small, unbalanced, or collected under overly controlled conditions [154,155,156,157,158]. For high-stakes applications, model performance must be tested with independent samples, external validation sites, realistic users, and clinically or operationally relevant thresholds [116,117,162]. Interpretability is also important. Users and regulators may need to understand why a device gives a positive result, an alarm, or a recommended action [158,162].

Machine learning is most useful when it is integrated with domain knowledge, quality-control rules, uncertainty estimation, and field validation [28,35,36,116,117,162].

### 7.4. Smartphone Apps and Cloud-Based Platforms

Smartphone applications and cloud-based platforms extend portable sensing from isolated measurement devices into digital decision systems [13,14,15,16,82,116]. A mobile application can guide the user through sample collection, reagent addition, incubation timing, image capture, sensor connection, result interpretation, and reporting [116,117]. It can also store data, record metadata, attach GPS coordinates, document operator identity, capture timestamps, and transmit results to clinicians, farm managers, environmental agencies, food-safety inspectors, or industrial dashboards [110,111,112,113,114,115,116,117]. Cloud platforms can aggregate measurements across sites and time, enabling surveillance, trend analysis, mapping, early warning, and coordinated decision-making [110,111,112,113,114,115,116,117].

These capabilities are valuable in many fields. In healthcare, connected point-of-care devices can support remote diagnosis, chronic disease monitoring, and integration with electronic health records [116,162]. In agriculture, portable and networked sensors can support fertilizer management, irrigation decisions, disease scouting, pesticide residue screening, and animal health monitoring [22,23,24,25,26,27,33,110,111,112,113,114,115]. In environmental monitoring, distributed water or air sensors can reveal contamination patterns and support timely intervention [22,23,24,110,111,112,113,114,115]. In industry, connected sensing can support process monitoring, worker safety, maintenance planning, and compliance documentation [110,115]. However, software design must consider usability, connectivity limitations, app maintenance, version control, device compatibility, data ownership, and workflow integration [116,117,118]. A technically accurate device may still fail if the application is confusing, the data cannot be transferred reliably, or the output does not align with the user’s decision-making process [116,117,118].

### 7.5. Decision Support and User-Centered Outputs

Decision support should be designed around the user, the application, and the consequence of action [116,117,118]. A physician may need a diagnostic category, a confidence interval, and a recommendation for confirmatory testing [3,38,116]. A farmer may need guidance on irrigation, fertilization, disease scouting, or harvest timing [22,23,33]. A food-safety inspector may need a pass/fail result, chain-of-custody record, and evidence for laboratory confirmation [25,26,27]. An industrial safety officer may need real-time alarms, exposure logs, and regulatory documentation [110,115]. A consumer may need simple guidance without technical details. Therefore, the most useful output of a portable sensor is not always a concentration value. In many cases, an interpretable decision category linked to a clear action is more valuable than a numerical signal [116,117,118].

Uncertainty communication is essential. Users should know whether a result is qualitative, semi-quantitative, or quantitative [163,164,165,166,167]. A device should report invalid tests, low-confidence results, out-of-range values, calibration problems, expired reagents, poor sample volume, or environmental conditions outside the validated range [116,117,163,164,165,166,167]. Overly precise numerical outputs can create false confidence when measurement uncertainty is large [163,164,165,166,167]. Well-designed decision support should communicate uncertainty in a way that is understandable and actionable, such as recommending retesting, repeating sampling, or sending the sample to a reference laboratory [116,117,118,163,164,165,166,167].

### 7.6. Data Integrity and Cybersecurity

Data integrity, privacy, cybersecurity, and traceability are increasingly important as portable sensing becomes connected [168,169,170]. Medical data, occupational exposure data, environmental monitoring records, food-safety results, and industrial process data can have personal, regulatory, commercial, or legal implications [116,117,168,169,170]. Connected systems should protect data at rest and in transit, authenticate users and devices, maintain audit trails, document calibration status, record timestamps, control permissions, and prevent unauthorized modification [168,169,170]. Secure cloud storage, encryption, version control, and chain-of-custody records are not optional additions when measurements affect health, safety, regulatory compliance, or industrial decisions [168,169,170].

Analytical trust now includes both chemical validity and digital trustworthiness [163,164,165,166,167,168,169,170]. A sensor result may be scientifically valid, but if the data record lacks traceability, calibration history, user identity, or protection against tampering, it may not be accepted in clinical, regulatory, or industrial settings [163,164,165,166,167,168,169,170]. Conversely, a secure digital system cannot compensate for poor sensing chemistry or weak validation [3,38,123]. The future of portable sensing will therefore depend on an integrated design in which signal processing, calibration, machine learning, software platforms, cybersecurity, uncertainty communication, and decision rules are developed alongside the physical sensing device [28,35,36,116,117,118,163,164,165,166,167,168,169,170].

## 8. Analytical Performance and Validation Criteria

Analytical validation is essential for transforming portable sensing systems from promising prototypes into trustworthy tools for real-world decision-making. A portable sensor may show impressive sensitivity, rapid response, or elegant device design in a laboratory demonstration, but these characteristics alone do not guarantee practical reliability [171,172,173,174,175]. Validation must connect performance claims to the device’s intended use [171,175]. A sensor designed for preliminary screening of contaminated water does not require the same validation pathway as a regulated clinical diagnostic device [22,23,24,171,172]. Yet both require evidence that the result is sufficiently reliable to support the decision it underpins [163,164,165,166,167,171]. Similarly, a wearable exposure monitor, a food-residue screening kit, a greenhouse nutrient sensor, and an industrial gas alarm must be evaluated according to different thresholds, users, matrices, environments, and error consequences [25,26,27,29,30,104,105,106,107].

Analytical performance criteria commonly include sensitivity, limit of detection, limit of quantification, dynamic range, selectivity, accuracy, precision, reproducibility, recovery, stability, response time, robustness, throughput, usability, and cost [171,172,173,174,175]. For portable sensing systems, additional system-level criteria are equally important. These include total sample-to-answer time, number of operator steps, power consumption, storage tolerance, environmental operating range, calibration frequency, device failure rate, data integrity, waste handling, and compatibility with real workflows [3,19,38,116,117,118]. Without rigorous and transparent validation, portable sensing risks producing attractive data that cannot be trusted in clinical practice, agriculture, food safety, environmental protection, industrial operation, or public safety [163,164,165,166,167,171].

### 8.1. Sensitivity and Limit of Detection

Sensitivity describes how strongly a sensor response changes with analyte concentration. At the same time, the limit of detection indicates the lowest concentration that can be reliably distinguished from the blank or background signal [171,172,173,174]. The limit of quantification defines the lowest concentration that can be measured with acceptable accuracy and precision [171,172,173,174]. These metrics are frequently emphasized in academic publications because they are easy to compare and often demonstrate the apparent strength of a new recognition chemistry or transduction method [173,174]. Dynamic range is also important because a sensor must cover the concentration interval relevant to its application [171,175].

However, extremely low detection limits are not always necessary or even useful. A nitrate sensor for farm management, a gas sensor for workplace safety, a food freshness indicator, or a chlorine sensor for water treatment only needs sensitivity aligned with practical decision thresholds [22,23,24,25,26,27]. Overemphasis on ultralow detection limits can distract from matrix tolerance, calibration stability, reproducibility, and usability [123,171,175]. A sensor that detects trace levels in clean buffer may still fail when used in blood, sweat, soil extract, wastewater, plant sap, food homogenate, or industrial effluent [22,23,24,25,26,27,29,30,123]. Therefore, sensitivity should be reported in relevant matrices and compared with the concentration range at which decisions must be made [171,175]. Practical validation should assess whether the sensor is sufficiently sensitive for its intended use, rather than whether it achieves the lowest possible concentration under ideal laboratory conditions [171,175].

### 8.2. Selectivity and Interference Resistance

Selectivity is the ability of a sensing system to distinguish the target analyte from chemically or biologically similar species and from unrelated interfering substances [171,175,176]. This criterion is crucial because real samples contain many compounds that may bind nonspecifically, react chemically, absorb or scatter light, foul electrodes, inhibit enzymes, affect pH, alter ionic strength, or produce overlapping signals [22,23,24,25,26,27,49,50,76,123]. A glucose sensor must resist interference from ascorbic acid, uric acid, acetaminophen, and other electroactive species [51,52,176]. A heavy-metal sensor must distinguish among competing ions [22,23,176]. A pathogen sensor must avoid cross-reactivity with related organisms [20,21,40,41,42]. A food-safety sensor must tolerate proteins, fats, pigments, salts, preservatives, and processing residues [25,26,27,135,136]. An environmental sensor must function despite the presence of humic substances, suspended solids, salinity, and variable organic matter [22,23,24,137,138,139].

Selectivity testing should therefore include realistic interferents at realistic concentrations [175,176]. It should not be limited to purified targets or a few simplified laboratory compounds [123,175,176]. Matrix effects should be assessed directly by testing real samples and, where appropriate, matrix-matched standards [123,135,136,137,138,139]. For portable systems, selectivity also includes resistance to fouling, nonspecific adsorption, background color, turbidity, humidity, temperature, and user handling [49,50,76,123,177,178]. A device that is selective in a controlled buffer system may not be selective in real field conditions [123,175,176]. Interference studies should be reported transparently, including the substances tested, their concentrations, the sample matrix, and their effects on the sensor response [175,176].

### 8.3. Accuracy, Precision, and Reproducibility

Accuracy, precision, and reproducibility determine whether a portable sensing result can be trusted [163,164,165,166,167,171,175,179,180,181,182]. Accuracy refers to closeness to the true value or to a validated reference method [163,164,165,166,167,171,175]. Precision refers to repeatability under the same conditions, such as repeated measurements by the same operator using the same device and sample type [179,180]. Reproducibility refers to agreement across devices, batches, operators, laboratories, days, and environmental conditions [179,180,181,182]. Portable sensors often show good repeatability in researchers’ hands but weaker reproducibility when accounting for fabrication variation, reagent aging, operator differences, and field conditions [123,175,179].

Validation should therefore include multiple devices, multiple production lots, multiple operators, and representative samples [175,179,180,181,182]. Recovery studies can show whether known amounts of analyte can be measured accurately in real matrices [175]. Comparison with reference laboratory methods is important when such methods exist, especially for clinical, food-safety, environmental, and regulatory applications [178,182]. Bias, agreement, confidence intervals, and classification performance should be reported, not only correlation coefficients [178,182]. A high correlation with a reference method does not necessarily mean acceptable agreement for decision-making [178]. The acceptable level of error depends on the intended use [171,175]. Screening devices may tolerate greater uncertainty if they are designed to identify samples that require confirmation, whereas diagnostic or regulatory devices require stronger evidence of accuracy and reproducibility [163,164,165,166,167,171].

### 8.4. Stability and Shelf Life

Stability and shelf life are critical for field deployment and commercial translation. A sensor may perform well immediately after fabrication but fail after storage, shipping, temperature cycling, exposure to humidity, or mechanical stress [183,184,185]. Enzymes may denature, antibodies may lose binding activity, nucleic acid reagents may degrade, nanoparticles may aggregate, electrodes may oxidize, membranes may dry or swell, and paper substrates may absorb moisture [53,54,140,141,142,143]. Reagent instability is especially important in hot and humid environments, where storage conditions are difficult to control, and cold-chain logistics may not be available [3,38,140,141,142,143].

Stability testing should include realistic storage temperatures, humidity levels, packaging formats, transportation conditions, vibration, light exposure, and time [183,184,185]. Accelerated aging studies can provide early estimates of shelf life, but real-time stability studies should support them, as accelerated conditions do not always recapitulate actual degradation pathways [183,184,185]. In-use stability is also important. A reusable sensor may drift during repeated measurements, while a wearable sensor may degrade during continuous contact with sweat, skin, motion, and biofouling [29,30,104,105,106,107]. Commercial portable systems require predictable shelf life and clear expiration criteria [183,184,185]. Packaging, desiccants, sealed cartridges, stabilizing additives, protective coatings, and self-check functions can improve stability, but their effectiveness must be validated [140,141,142,143,183,184,185,186,187,188,189].

### 8.5. Response Time and Throughput

Response time and throughput strongly influence the operational value of portable sensing. Response time should not be defined only as the time required for the sensing surface to produce a signal after exposure to the prepared sample [19,38,116]. The more relevant metric is total sample-to-answer time, which includes sample collection, preparation, extraction, filtration, reaction, incubation, amplification, signal acquisition, data processing, interpretation, cleaning, and reporting [19,38,116,123]. A sensor with a one-minute signal response may still be impractical if sample preparation requires thirty minutes of manual handling [123,124].

Different applications require different time scales. Emergency diagnostics, industrial gas alarms, occupational safety monitors, and security screening may require results within seconds or minutes [19,38,45]. Food inspection, environmental surveillance, and agricultural testing may tolerate longer times if the result avoids laboratory transport or enables same-day action [22,23,24,25,26,27,33]. Nucleic acid assays may require amplification time but can still be valuable when they provide accurate on-site pathogen detection [20,21,59,60,61,62,124,128]. Throughput matters when many samples must be screened, such as in food-processing plants, outbreak testing, water-quality surveys, greenhouse crop monitoring, or industrial quality control [19,25,26,27,33]. Portable systems should therefore report both single-sample response time and practical throughput under realistic operating conditions [19,38,116,175].

### 8.6. Usability and Operator Independence

Usability is often underestimated in validation, yet it strongly determines whether a portable sensing system succeeds outside the laboratory [116,117,118,190,191,192]. A practical device should minimize pipetting, manual washing, timing errors, ambiguous color interpretation, complex calibration, sample-transfer steps, and exposure to hazardous materials [116,117,118]. Instructions should be clear, results should be readable, and failure modes should be detected automatically when possible [116,117,118,190,191,192]. The device should guide users through sample addition, incubation, measurement, interpretation, and disposal [116,117,118]. For many applications, the user may be a patient, farmer, food inspector, wastewater operator, factory technician, emergency responder, or consumer rather than an analytical chemist [116,117,118].

Human factors testing can reveal problems that are invisible during laboratory validation [117,118,190,191,192]. A prototype operated by its inventors may perform well because the users understand every design detail. The same device may fail when used by non-specialists under time pressure, in poor lighting, in high humidity, or in stressful field conditions [117,118,190,191,192]. Validation should therefore include representative users, realistic settings, and observation of user errors [117,118,190,191,192]. Operator independence does not mean eliminating all training, but it does mean reducing opportunities for mistakes and translating analytical signals into clear decisions [116,117,118]. Usability should be treated as a performance criterion, not merely as a product-design preference [117,118,190,191,192].

### 8.7. Tiered Validation and Transparent Reporting

Validation should be staged according to development maturity [171,175,193,194,195,196,197]. Early-stage validation may test the analytical principle using standards and controlled samples. Intermediate validation should include spiked matrices, real samples, multiple devices, environmental variation, interference studies, and preliminary user testing [175,176,179]. Advanced validation should compare results with reference methods, evaluate field operation, assess stability and user error, and demonstrate performance under intended-use conditions [178,182,183,184,185,190,191,192]. For regulated applications, validation must follow relevant standards, quality systems, and regulatory expectations [171,172,179,182,183,184,185,198]. This tiered approach helps prevent premature claims while supporting systematic translation from proof of concept to practical deployment [171,175,193,194,195,196,197].

Transparent reporting is essential for comparing sensors and accelerating adoption [193,194,195,196,197]. Reports should include the number of samples, devices, and fabrication batches; the number and types of operators; matrix composition; environmental conditions; calibration method; reference method; statistical analysis; confidence intervals; failure rates; and exclusion criteria [175,193,194,195,196,197]. Performance should be reported honestly, including limitations and conditions under which the device is not validated [193,194,195,196,197]. Portable sensing will gain practical trust only when validation demonstrates that the complete system can deliver sufficiently accurate, timely, stable, interpretable, and reproducible information at the location where that information is needed [3,38,171,175].

**Figure 5:**
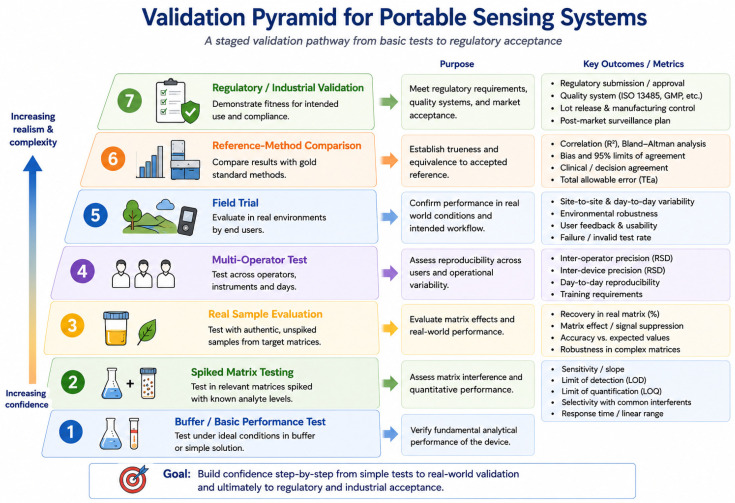
Validation pyramid for portable sensing systems.

The Validation pyramid for portable sensing systems is shown in Figure 5. This figure shows a staged validation model: buffer test → spiked matrix → real sample → multi-operator test → field trial → reference-method comparison → regulatory/industrial validation.

## 9. Design Challenges and Technical Limitations

Portable sensing systems face design challenges that arise from miniaturization, environmental exposure, simplified operation, cost constraints, and the need for reliable use by non-specialists [19,38,116,117,199,200,201,202]. These limitations should not be viewed only as weaknesses. They are the engineering constraints that define the real problem of portable analysis. A successful device does not merely miniaturize a laboratory method; it balances sensitivity, selectivity, stability, power consumption, manufacturability, usability, durability, documentation, and regulatory acceptance [171,175,199,200,201,202]. The central challenge is to build systems that remain reliable when used by real people, with real samples, under real operating conditions, over a realistic product lifetime [116,117,118,123,175]. Many prototypes demonstrate excellent detection in controlled settings, but practical adoption depends on whether the complete workflow can function outside the laboratory [19,38,200,201,202].

### 9.1. Sensitivity Versus Portability

The first major challenge is the trade-off between analytical sensitivity and practical portability. High sensitivity may require complex optics, low-noise electronics, long incubation, sample enrichment, nucleic acid amplification, temperature control, multiple washing steps, or precise fluid manipulation [45,59,60,61,62,124,199,200,201,202]. These requirements may increase device size, cost, power demand, user burden, and failure risk [19,38,200,201,202]. Portability, in contrast, requires small size, low power consumption, simple operation, fast results, rugged construction, and minimal manual handling [3,38,116,117]. The optimal design therefore depends on the intended use. A device for trace-level toxin detection may justify enrichment and longer processing time. In contrast, a field screening device for farm management, occupational safety, or food freshness may benefit more from speed, ruggedness, and moderate sensitivity [22,23,24,25,26,27,33].

In many practical contexts, a robust device with sufficient sensitivity is preferable to a fragile device with exceptional detection limits [171,175,199,200,201,202]. The goal should not always be laboratory-equivalent performance, but decision-relevant performance. A portable sensor should be sensitive enough to detect the concentration range relevant to the intended action [171,175]. When the required decision threshold is far above the limit of detection, further lowering the detection limit may not improve practical value [173,174,175]. Instead, design effort may be better directed toward matrix tolerance, reproducibility, calibration stability, and ease of use [123,175,199,200,201,202].

### 9.2. Reproducibility of Sensor Fabrication

Reproducibility in sensor fabrication is one of the most serious barriers to translating academic prototypes into industrial products [77,79,203,204,205,206,207]. Many laboratory prototypes rely on manual electrode modification, drop casting, hand-cut paper or microfluidic structures, small-batch nanomaterial synthesis, manually immobilized enzymes or antibodies, and individually adjusted measurement conditions [53,54,96,97,98,203,204,205,206,207]. These methods can produce excellent individual devices but often lead to large variation across batches, operators, and laboratories [175,179,180,181,182]. Such variation affects calibration, response time, background signal, selectivity, and shelf life [151,152,153,175,183,184,185].

Industrial translation requires controlled materials, scalable fabrication, in-process quality assurance, packaging, lot release testing, and defined acceptance criteria [79,171,175,203,204,205,206,207]. Screen printing, roll-to-roll manufacturing, injection molding, laser patterning, photolithography, automated reagent deposition, and standardized surface chemistry may improve scalability [77,79,203,204,205,206,207]. However, manufacturability must be considered early in design rather than after the sensing concept has already been optimized using non-scalable methods [19,38,117,119]. A recognition layer that performs well only when prepared manually by an expert may not be suitable for commercial deployment [206,207,208]. Therefore, portable sensor development should include batch-to-batch testing, manufacturing tolerance analysis, and quality-control procedures from an early stage [175,179,180,181,182,183,184,185,203,204,205,206,207].

### 9.3. Biofouling and Sensor Drift

Biofouling and sensor drift limit both repeated-use and long-term portable sensing [199,209,210,211,212]. Real samples often contain proteins, cells, bacteria, organic matter, fats, particles, salts, and reactive compounds that adsorb to sensor surfaces, block recognition sites, alter optical properties, change electrode behavior, or disrupt fluid flow [22,23,24,25,26,27,49,50,76,123]. In biological samples, protein adsorption and cell adhesion can reduce sensitivity or produce nonspecific responses [125,126,127,209,210,211,212]. In environmental and food samples, organic matter, pigments, oils, and suspended solids can foul surfaces and interfere with signal generation [22,23,24,25,26,27,135,136,137,138,139]. In wearable devices, sweat components, skin debris, motion, and microbial growth may gradually change sensor response [104,105,106,107,132,133,134].

Sensor drift may result from electrode degradation, reagent depletion, enzyme denaturation, antibody instability, nanoparticle aggregation, membrane swelling, temperature fluctuation, mechanical stress, reference electrode instability, or biological degradation [49,50,53,54,140,141,142,143,160,161]. Continuous monitoring systems require drift management more urgently than single-use tests because small baseline changes can accumulate over time and be misinterpreted as biological or chemical trends [29,30,104,105,106,107,199]. Strategies to reduce fouling and drift include antifouling coatings, protective membranes, disposable sensing elements, reference channels, periodic calibration, self-cleaning surfaces, surface regeneration, signal normalization, and drift-correction algorithms [76,151,152,153,160,161,199,209,210,211,212]. These strategies must be validated in real matrices and over realistic operating times [123,175,199].

### 9.4. Power Supply and Device Durability

Power supply and durability are practical constraints that strongly influence user trust and adoption [104,105,106,107,213,214,215,216]. Portable devices may use coin cells, rechargeable batteries, replaceable battery packs, energy harvesting, or smartphone power [13,14,15,16,213,214]. Power consumption depends on the sensor type, signal acquisition electronics, pumps, valves, heaters, optics, wireless communication, processors, displays, and data storage [19,38,213,214]. Nucleic acid amplification, thermal control, fluorescence excitation, and wireless transmission can significantly increase energy demand [20,21,59,60,61,62,213,214]. Wearable devices must be lightweight, safe, flexible, and comfortable, while field instruments must operate for long periods without frequent charging [29,30,104,105,106,107,213,214,215,216].

Durability is equally important. Field devices may be exposed to dust, rain, humidity, sunlight, vibration, impact, temperature extremes, corrosive chemicals, and rough handling [19,38,183,184,185]. Wearable devices must tolerate bending, stretching, sweat, washing, and skin contact [104,105,106,107,215,216]. Industrial devices may need chemical resistance, electromagnetic shielding, explosion-safe design, and rugged housings [110,115]. Durability testing should not be treated as a final packaging exercise. It should be part of development from the beginning, because housing, fluidics, connectors, seals, buttons, screens, cartridges, and power systems can determine whether the analytical function remains usable in practice [19,38,183,184,185,213,214,215,216].

### 9.5. Incomplete Integration of Portable Workflows

A common limitation of portable sensing research is incomplete integration [19,38,116,117,123]. Many prototypes are described as portable because their sensor elements are small. However, the complete workflow still requires external equipment, manual pipetting, laboratory centrifuges, desktop potentiostats, microscopes, controlled incubation, refrigerated reagents, washing steps, or expert interpretation [19,32,123,200,201,202]. In these cases, the device may be physically small but not truly field-deployable. True portability requires that all necessary steps, consumables, reagents, sample handling, waste containment, calibration, signal reading, and interpretation be compatible with use outside the laboratory [19,38,116,117].

This issue is especially important for nucleic acid tests, immunoassays, and sensors for complex matrices [20,21,55,56,57,123,128,200,201,202]. Sample extraction, lysis, filtration, washing, amplification, reagent storage, and contamination control may dominate practical complexity [123,124,125,126,127,128,217]. A highly sensitive recognition reaction may have little field value if the preparation steps require laboratory infrastructure [123,200,201,202]. Complete integration often requires cartridges, sealed reagent storage, passive or automated fluid movement, simplified user steps, and built-in quality checks [19,38,124,128]. The design objective should be sample-to-answer operation whenever possible [19,124,128,200,201,202].

### 9.6. Cost, Manufacturability, and Adoption

Cost is both a technical and economic limitation. A disposable sensor must be inexpensive enough for routine use, while a reusable reader must justify the cost of purchase, calibration, maintenance, software updates, and training [200,201,218,219,220,221,222]. Expensive nanomaterials, antibodies, microfabricated components, specialized cartridges, or proprietary readers may limit adoption, especially in agriculture, resource-limited healthcare, environmental monitoring, and large-scale screening [3,7,8,38,200,201]. Cost should be evaluated as total cost per useful decision, not merely the price of the sensing element [200,201,218,219,220,221,222]. This total includes the device, consumables, labor, training, calibration, maintenance, invalid tests, false results, data management, waste disposal, and confirmatory testing [116,117,200,201].

Another challenge is the mismatch between academic novelty and user need [116,117,118,200,201]. A complex nanostructure, multi-step assay, or sophisticated signal-processing method may be scientifically publishable but practically unnecessary if it increases cost or complexity without improving the decision [117,200,201]. Conversely, improvements in sampling, packaging, calibration, reagent stability, or user instructions may have major practical value even if they appear less novel scientifically [19,38,116,117,118]. Successful translation requires early attention to real users, workflows, price constraints, supply chains, and maintenance capacity [116,117,118,119,200,201,218,219,220,221,222].

### 9.7. Standardization and Regulatory Acceptance

Standardization and regulatory acceptance remain difficult because portable sensing systems operate across diverse domains, including medical diagnostics, environmental monitoring, food safety, industrial quality control, forensic investigation, and occupational safety [171,175,198,223,224,225,226]. Each domain has different validation expectations, reference methods, documentation requirements, risk levels, and regulatory pathways [171,172,173,174,175,176,177,178,179,180,181,182,183,184,185,186,187,188,189,190,191,192,193,194,195,196,197,198,223,224,225,226]. A device intended for clinical decision-making must demonstrate clinical performance, not only analytical detection [171,193,194,195,196,197,198,223,224,225,226]. A device intended for regulatory environmental measurement must demonstrate traceability, quality assurance, and comparability with accepted methods [163,164,165,166,167,175]. A food-safety screening tool must define how positive results are confirmed and how records are maintained [25,26,27,175].

Acceptance depends on evidence, documentation, traceability, calibration records, quality systems, and alignment with existing standards [163,164,165,166,167,171,172,173,174,175,176,177,178,179,180,181,182,183,184,185,186,187,188,189,190,191,192,193,194,195,196,197,198,223,224,225,226]. Standardized reporting of sensor performance would improve comparison across studies and accelerate translation [193,194,195,196,197]. Important information includes sample matrix, number of devices, number of batches, operator variation, environmental conditions, calibration method, reference method, failure rate, stability data, and intended-use limitations [175,193,194,195,196,197]. Without this transparency, promising devices remain difficult to compare, reproduce, regulate, and adopt [171,175,193,194,195,196,197].

Table 5 matches key limitations with design responses: biofouling, drift, power supply, device durability, incomplete workflow integration, manufacturability, regulatory acceptance, and cost control.

In summary, the major limitations of portable sensing extend beyond sensitivity and miniaturization. They include fabrication reproducibility, biofouling, drift, power supply, durability, workflow integration, cost, usability, standardization, and regulatory acceptance [220,221,222,223,224,225,226]. Addressing these challenges requires a system-level design philosophy in which chemistry, engineering, manufacturing, data processing, packaging, human factors, and validation are developed together [19,38,116,117,118,171,175]. Only then can portable sensing systems move from attractive prototypes to dependable tools for real-world biological and chemical analyses [220,221,222,223,224,225,226].

## 10. Gap Between Academic and Industrial Application

The gap between academic research and industrial application is one of the most important issues in portable sensing systems. Many studies report impressive proof-of-concept performance, including low detection limits, novel nanomaterials, elegant recognition mechanisms, rapid responses, and successful detection in controlled laboratory samples [4,19,38,199,200,201,202]. However, only a small portion of these prototypes become widely adopted products [4,200,201,227,228]. This gap is not simply a commercialization failure. It reflects a mismatch between academic and industrial evaluation criteria. Academic studies often emphasize novelty, sensitivity, selectivity, mechanistic insight, and publication value [171,175,199]. Industrial users ask whether the system can be manufactured reproducibly, stored safely, operated by non-specialists, maintained under field conditions, integrated into workflows, connected to data systems, documented for quality assurance, and trusted for clinical, economic, environmental, legal, or safety decisions [116,117,118,119,171,175,200,201,225,226].

A sensor that produces an excellent publication may still be unsuitable for deployment if it works only in a clean buffer, requires freshly prepared reagents, depends on manual laboratory steps, or cannot be produced consistently [123,175,199,200]. Industrial translation requires a shift from sensor-centered research to system-centered development [4,19,38,119,227]. The key question is not only whether a target can be detected, but whether the complete system can deliver reliable, timely, affordable, interpretable, and actionable information under intended-use conditions [3,38,116,117,118,200,201]. This requires attention to sampling, preparation, recognition chemistry, signal transduction, packaging, calibration, software, data integrity, usability, manufacturability, maintenance, regulation, and market value [19,38,116,117,118,119,171,175,225,226].

### 10.1. From Laboratory Prototype to Field-Ready Product

The transition from laboratory prototype to field-ready product requires a change in design priorities [4,19,38,227,228]. In the laboratory, temperature, humidity, sample composition, reagent handling, incubation time, and measurement conditions are controlled. Samples are prepared carefully, operators are trained, and devices may be tested soon after fabrication [123,175]. In the field, samples may be heterogeneous, users may have limited training, reagents may have been stored for months, and devices may be exposed to dust, moisture, vibration, sunlight, heat, cold, and rough handling [19,38,183,184,185,200]. A field-ready product must tolerate these variations or detect when conditions fall outside its valid operating range [116,117,171,175].

Robustness is therefore part of analytical performance [175,186,187,188,189]. A device with an extremely low detection limit under ideal conditions but poor field stability is less useful than a moderately sensitive device that performs reliably in real workflows [171,175,199]. Field readiness also requires clear instructions, protected reagents, rugged packaging, simple sample introduction, secure waste containment, understandable outputs, and quality-control checks [19,38,116,117,118]. A portable sensing system should be evaluated as a field-operable analytical tool, not merely as a laboratory component [4,19,38,200].

### 10.2. Proof-of-Concept Performance and Practical Reliability

Proof-of-concept performance differs greatly from practical reliability [171,175,199,200,201,202]. A paper may report a low detection limit using one device, a small number of spiked samples, or an optimized buffer system. Such results demonstrate feasibility but do not prove that the system can operate reliably in daily use [123,175,199]. Practical reliability requires performance across real samples, device lots, operators, environments, and storage periods [175,179,180,181,182,183,184,185]. It also requires knowledge of false-positive rates, false-negative rates, invalid-test rates, confidence intervals, and failure modes [171,172,173,174,175,176,177,178,193,194,195,196,197].

Field users need to know when to trust a result, repeat the measurement, or send the sample for confirmatory laboratory analysis [116,117,118,171,175]. This requires validation with negative controls, positive controls, blind samples, real matrices, and realistic deployment protocols [175,176,177,178,179,180,181,182,193,194,195,196,197]. A portable pathogen test must work when sample collection is imperfect, inhibitors are present, pathogen levels are low, and there is a risk of contamination [20,21,123,124,125,126,127,128,217]. A gas sensor must be tested against humidity, temperature variation, gas mixtures, and cross-sensitive compounds [47,159,160,161]. Practical reliability is therefore broader and more demanding than proof-of-concept success [175,199,200,201,202].

### 10.3. Sample Preparation as the Missing Link

Sample preparation is often the missing link in commercialization [123,124,128]. Many academic sensors require centrifugation, extraction, dilution, filtration, pH adjustment, incubation, washing, reagent mixing, or careful pipetting before detection [123,124,125,126,127,128,217]. These steps may be acceptable in research laboratories but are major barriers in field use [19,38,123,200]. Each manual step adds time, training demand, contamination risk, measurement variation, user error, and cost [117,123,200]. A sensor is not truly portable if its sample-preparation workflow remains laboratory-based [19,38,123].

Industrial and clinical users usually prefer sample-to-answer operation, in which a raw or minimally processed sample produces an interpretable result with minimal handling [19,124,128,200]. Achieving this requires integration of sample collection, pretreatment, reagent storage, fluid control, reaction timing, waste containment, and detection [19,38,123,124]. Nucleic acid tests must integrate lysis, extraction, inhibitor removal, amplification, contamination control, and detection [20,21,59,60,61,62,123,124,125,126,127,128,217]. Immunoassays must simplify or automate the washing and labeling steps [55,56,57]. Environmental and food samples require practical extraction and filtration [24,25,26,27,135,136]. Commercial development should treat sample preparation as a core design problem, not as an accessory after sensor optimization [123,124,128].

### 10.4. Reproducibility and Batch-to-Batch Variation

Reproducibility and batch-to-batch variation are major barriers to industrial translation [175,179,180,181,182,203,204,205,206,207]. Academic prototypes often use hand-prepared nanomaterial films, manually functionalized electrodes, drop-cast reagents, small-batch polymer films, or laboratory-fabricated microchannels [53,54,77,96,97,98,203,204,205,206,207]. These methods may produce excellent individual devices but large variation across batches [175,179,180,181,182]. Enzyme activity, antibody loading, aptamer density, nanoparticle size, electrode roughness, polymer morphology, membrane thickness, and reagent distribution can all affect sensor response [53,54,63,64,65,203,204,205,206,207]. If these variables are not controlled, each device may require a different calibration curve [151,152,153,175].

Industrial products require specifications, process controls, acceptance criteria, lot-release testing, traceable materials, and quality assurance [171,175,179,180,181,182,183,184,185,225]. Researchers can support translation by reporting device-to-device variation, fabrication yield, batch reproducibility, storage conditions, and failure rates rather than best-case results alone [175,179,180,181,182,183,184,185]. Design for manufacturability should begin early [119,203,204,205,206,207]. Screen printing, roll-to-roll processing, injection molding, automated dispensing, laser patterning, standardized surface chemistry, and controlled reagent deposition may improve scalability if they are compatible with the recognition chemistry and intended performance [77,79,203,204,205,206,207,229,230].

### 10.5. Stability, Shelf Life, and Storage Conditions

Stability, shelf life, and storage tolerance determine whether a portable sensor can be distributed, stored, and used when needed [183,184,185]. A device that works only immediately after fabrication is unsuitable for most markets [183,184,185,200]. Enzymes may denature, antibodies may lose affinity, nucleic acid reagents may degrade, nanoparticles may aggregate, electrodes may oxidize, membranes may dry or swell, and paper substrates may absorb moisture [53,54,140,141,142,143,183,184,185]. These problems are especially severe in tropical climates, field stations, rural clinics, farms, food-processing facilities, and resource-limited regions without reliable cold storage [3,38,140,141,142,143,200,201].

Packaging is therefore part of the analytical system [19,38,183,184,185]. Desiccants, oxygen barriers, foil pouches, stabilizing additives, lyophilized reagents, sealed cartridges, thermal protection, and humidity indicators may be needed to maintain performance [140,141,142,143,183,184,185]. Stability studies should include realistic supply-chain conditions such as shipping vibration, temperature cycling, humidity, light exposure, and long-term storage [183,184,185]. Accelerated aging provides early estimates, but real-time stability testing is still needed because degradation pathways may differ [183,184,185]. Industrial users need predictable shelf life, expiration criteria, and storage instructions [183,184,185,225].

### 10.6. Biofouling, Sensor Drift, and Long-Term Operation

Biofouling, sensor drift, and long-term operation are especially important for wearable, environmental, and industrial sensors [199,209,210,211,212]. A wearable sensor may operate for hours or days in contact with sweat, skin, motion, and microorganisms [104,105,106,107,132,133,134]. An environmental sensor may be deployed in dirty water containing organic matter, particles, microorganisms, and variable salinity [22,23,24,137,138,139]. An industrial sensor may face solvents, oils, dust, corrosive vapors, temperature swings, or high ionic strength [22,23,24]. Under these conditions, performance may decline through fouling, poisoning, reagent depletion, mechanical wear, electrode degradation, baseline drift, or membrane changes [49,50,76,160,161,199].

Performance decline should be measured directly rather than assumed negligible [175,183,184,185,199]. Disposable sensing elements, replaceable cartridges, antifouling coatings, protective membranes, self-cleaning surfaces, reference channels, calibration checks, and drift-correction algorithms can reduce these problems, but each adds cost or complexity [76,151,152,153,160,161,199,209,210,211,212]. Continuous monitoring systems require stronger drift management than single-use tests because gradual baseline shifts may be misinterpreted as real changes in the target [29,30,104,105,106,107,199]. Long-term deployment also requires maintenance plans, replacement schedules, self-diagnostics, and criteria for excluding invalid data [116,117,159,160,161].

### 10.7. Calibration Transfer and Inter-Device Consistency

Calibration transfer and inter-device consistency are central industrial issues [151,152,153]. A calibration model built on one device may not apply to another if electrode properties, optical geometry, reagent distribution, surface chemistry, or electronics differ [151,152,153,175]. Smartphone-based systems exhibit additional variability due to camera characteristics, automatic exposure, color processing, lighting conditions, and software versions [13,14,15,16,82]. Wearables may vary due to skin contact, sweat rate, movement, and placement [104,105,106,107,132,133,134]. Networked environmental sensors may vary because of temperature, humidity, fouling, and sensor aging [110,111,112,113,114,115,159,160,161].

Standardized manufacturing, internal references, onboard controls, environmental sensors, ratiometric signals, adaptive algorithms, and field verification can improve calibration transfer [13,14,15,16,80,81,82,151,152,153]. However, these strategies must be validated [175,179,180,181,182,183,184,185]. Industrial products must specify calibration frequency, acceptance limits, correction methods, and procedures for recalibration or replacement [163,164,165,166,167,175]. For connected sensor networks, consistency is essential because data from many devices are combined for trend analysis, alarms, and spatial mapping [110,111,112,113,114,115]. Without calibration transfer, large datasets may appear precise while hiding systematic bias [151,152,153,159,160,161].

### 10.8. Accuracy Compared with Standard Laboratory Methods

Accuracy compared with accepted reference methods is necessary for trust [163,164,165,166,167,171,175,177,178]. Reference methods such as chromatography, mass spectrometry, laboratory immunoassays, certified electrochemical analyzers, culture-based tests, and validated molecular methods provide benchmarks for portable devices [171,175,177,178]. Comparison should use real samples across the intended concentration range, not only spiked standards under ideal conditions [175,177,178]. The agreement should be evaluated using methods appropriate to the application [177,178,182].

Correlation alone is insufficient because two methods may correlate while showing systematic bias that affects decisions [178]. Depending on the use case, Bland–Altman analysis, recovery studies, confusion matrices, sensitivity and specificity estimates, decision-threshold analysis, and uncertainty evaluation may be needed [163,164,165,166,167,177,178,193,194,195,196,197]. Clinical diagnostics must demonstrate clinical sensitivity and specificity in relevant populations [193,194,195,196,197]. Food-safety screening must show how positive results relate to confirmation [25,26,27,175]. Environmental sensors must demonstrate comparability, traceability, and stability [163,164,165,166,167,175]. Reference-method comparison identifies bias, establishes credibility, and clarifies whether the portable device is a screening, monitoring, or confirmatory tool [175,177,178].

### 10.9. User-Centered Design and Operator Independence

User-centered design and operator independence strongly influence adoption [116,117,118,190,191,192]. Industrial users need simple procedures, clear interpretation, low training demand, resistance to user error, and compatibility with field conditions [116,117,118]. A device may fail commercially because it requires too many steps, produces ambiguous results, takes too long, needs delicate handling, or does not fit records and workflows [117,118,200]. The intended user may be a nurse, farmer, veterinarian, food inspector, wastewater operator, factory technician, emergency responder, or consumer rather than an analytical chemist [116,117,118].

Usability testing should involve representative users and realistic environments [117,118,190,191,192]. Instructions, sample tools, timers, packaging, app interfaces, error messages, protective-equipment compatibility, and waste disposal are part of the analytical system [116,117,118,190,191,192]. A prototype operated by its inventors may perform well, but this does not prove non-specialists can obtain reliable results [117,118]. Operator independence requires clear workflows, minimized manual steps, built-in checks, intuitive interpretation, and meaningful outputs [116,117,118]. In many applications, the best output is not a raw concentration value but a decision category, warning, or recommended action [116,117,118].

### 10.10. Manufacturing Scalability and Cost Control

Manufacturing scalability and cost control must be considered early [119,203,204,205,206,207,229,230]. Academic devices may use expensive materials, rare nanostructures, slow fabrication methods, fragile biomolecules, or specialized equipment because only a few prototypes are needed [7,8,53,54]. Industrial production requires stable supply chains, automated assembly, quality control, packaging, sterilization when needed, lot tracking, documentation, and cost-effective components [171,175,225]. A device that is scientifically elegant but too expensive or difficult to manufacture will not be adopted [200,201,218,219,220,221,222].

Cost should be evaluated as total cost per useful decision rather than the price of the sensor element alone [200,201,218,219,220,221,222]. This includes the reader, disposable cartridge, reagents, sampling tools, labor, training, maintenance, calibration, software, data storage, invalid tests, false results, confirmation, service, and waste disposal [116,117,200,201]. In some settings, a more expensive device may be justified if it prevents costly failures, reduces labor, shortens decision time, or improves safety [218,219,220,221,222]. In others, even a strong device may fail if the economic benefit is unclear [200,201]. Design-for-manufacture and design-for-cost should be treated as research criteria [119,203,204,205,206,207,229,230].

### 10.11. Integration of Hardware, Software, Data Processing, and Communication

A portable sensing product is often a cyber–physical analytical system rather than a simple sensor [110,111,112,113,114,115,116,117,168,169,170]. It may include a disposable cartridge, a reusable reader, embedded electronics, a power supply, firmware, a mobile app, a cloud server, a database, a calibration model, a dashboard, and a decision-support interface [13,14,15,16,82,116,117]. Failure in any component can compromise the result [116,117,168,169,170]. Stable recognition chemistry cannot compensate for unreliable Bluetooth connection, poor battery life, confusing software, unvalidated algorithms, or insecure data storage [116,117,118,162,168,169,170].

Integration determines whether the sensor becomes a complete analytical product [19,38,116,117,118]. Hardware must support reliable signal acquisition, environmental compensation, and rugged operation [144,145,146,147,213,214,215,216]. Software must guide users, process signals, manage calibration, detect errors, and present interpretable outputs [116,117,144,145,146,147,148,149,150,151,152,153,154,155,156,157,158,159,160,161,162]. Communication systems must transmit data securely and reliably [168,169,170]. Cloud platforms may support longitudinal monitoring, geotagging, surveillance, and decision support [110,111,112,113,114,115,116,117]. Firmware and app updates must be controlled because software changes may affect interpretation [116,117,168,169,170]. Industrial development therefore requires coordinated engineering across chemistry, mechanics, electronics, software, data science, cybersecurity, and human factors [116,117,118,168,169,170,231,232,233].

### 10.12. Data Quality, Cybersecurity, and Traceability

As portable sensing becomes connected, digital trust becomes inseparable from analytical trust [168,169,170,231,232,233]. Data integrity, privacy, secure transmission, audit trails, timestamps, calibration records, user permissions, chain-of-custody documentation, and decision logs are increasingly important [168,169,170,231,232,233]. Medical data may be sensitive. Occupational exposure data may affect worker rights and employer responsibilities. Food-safety and environmental data may have regulatory implications. Industrial process data may reveal proprietary information [168,169,170,231,232,233].

Connected devices must protect data during storage and transmission, authenticate users and devices, prevent unauthorized modification, and document calibration status [168,169,170,231,232,233]. A sensor result may be analytically valid but unacceptable for regulated or industrial use if the data record lacks traceability [163,164,165,166,167,168,169,170]. Conversely, secure digital records cannot compensate for poor chemistry or weak validation [3,38,123,175]. Reliable portable sensing requires both valid measurement and trustworthy data management [163,164,165,166,167,168,169,170]. Cybersecurity should therefore be considered during design rather than added after deployment [168,169,170,231,232,233].

### 10.13. Regulatory Approval, Certification, and Standardization

Regulatory approval, certification, and standardization differ across domains but are unavoidable for serious deployment [171,175,198,223,224,225,226,234,235,236,237,238,239]. Clinical devices may require demonstration of clinical sensitivity, specificity, risk control, quality management, and usability [171,193,194,195,196,197,198,223,224,225,226,234,235,236,237,238,239]. Food-safety and environmental devices may need to be compared with accepted methods, recognized by agencies, traceable to calibration, and subject to quality assurance [163,164,165,166,167,175]. Industrial safety devices may need to conform to occupational, electrical, explosion-safety, or environmental standards. Forensic and security devices may require chain-of-custody procedures and confirmatory pathways [163,164,165,166,167,175].

Early engagement with regulatory expectations can prevent designs from failing despite good laboratory performance [233,234,235,236,237,238,239]. Regulation requires a clear intended use, documented evidence, risk analysis, manufacturing controls, labeling, user instructions, quality systems, and, where appropriate, post-market monitoring [223,224,225,226,227,228,229,230,231,232,233,234,235,236,237,238,239]. Standardized reporting would improve comparison across studies and accelerate translation [193,194,195,196,197]. Reports should include matrices, device numbers, batch numbers, environmental conditions, operator variation, calibration methods, reference methods, failure rates, storage stability, and intended-use limitations [175,193,194,195,196,197].

### 10.14. Market Needs, Business Models, and Adoption Barriers

Market need determines whether a portable sensing system creates value [200,201,218,219,220,221,222]. A device should solve a real problem, reduce cost or labor, shorten decision time, improve safety, increase access, simplify reporting, or enable previously impossible decisions [218,219,220,221,222,240,241,242,243]. Some sensors are scientifically interesting but economically unnecessary because existing methods are already cheap, fast, and sufficient. Others may be valuable even with moderate accuracy because they provide immediate screening in underserved settings or reduce the consequences of delay [200,201,218,219,220,221,222].

Adoption depends on trust, training, maintenance, procurement, reimbursement, service models, supply chains, and compatibility with procedures [200,201,218,219,220,221,222,240,241,242,243]. In healthcare, reimbursement and workflow integration may determine adoption [221,222,240,241,242,243]. In agriculture, cost per test and connection to management decisions may be decisive [22,23,33]. In food safety, inspection compatibility and confirmatory testing are essential [25,26,27]. In industrial safety, reliability, alarm clarity, maintenance, and regulatory acceptance matter [110,115]. Business models must consider whether revenue comes from devices, cartridges, software subscriptions, data services, maintenance contracts, or integrated monitoring solutions [200,201,218,219,220,221,222,240,241,242,243].

Table 6 compares academic proof-of-concept criteria with industrial deployment requirements. This is especially useful because the paper strongly emphasizes the mismatch between publication-oriented novelty and product-level reliability.

### 10.15. Bridging Academic Research and Industrial Translation

Bridging academic research and industrial translation requires early industry collaboration, field validation, manufacturability testing, reference-method comparison, usability trials, standardized reporting, and interdisciplinary teams [4,19,38,116,117,118,119,200,201]. Researchers should report not only analytical novelty but also real-sample performance, device yield, storage stability, matrix effects, batch variation, user steps, total assay time, failure rates, and cost considerations [175,179,180,181,182,183,184,185,193,194,195,196,197]. These details make the literature more useful for engineers, manufacturers, regulators, investors, and end users [4,19,38,200,201].

A translational roadmap can be imagined in stages. The first stage demonstrates the sensing principle under controlled conditions [171,175]. The second validates the principle in relevant matrices [123,175]. The third integrates sample preparation, reagent storage, packaging, and user interface [19,38,116,123,124]. The fourth compares real-sample results with reference methods [175,177,178]. The fifth tests field operation with representative users [116,117,118,190,191,192]. The sixth develops scalable manufacturing, quality control, and calibration transfer [151,152,153,203,204,205,206,207,225]. The seventh addresses regulatory, service, market, and post-deployment requirements [200,201,218,219,220,221,222,223,224,225,226,234,235,236,237,238,239,240,241,242,243]. Many academic projects stop at the first or second stage, leaving the most difficult engineering and adoption work incomplete [4,19,38,200,201].

The future of portable sensing depends on recognizing that the industrial product is not only a sensing material or a small device. It is a complete analytical system embedded in a workflow [19,38,116,117,118,119]. Successful translation requires that research questions include deployment constraints from the beginning [4,19,38,200,201]. When chemistry, engineering, manufacturing, data processing, human factors, regulation, and market value are integrated, portable sensing systems can move beyond attractive prototypes to become dependable tools for biological and chemical analyses in real-world settings [241,242,243].

## 11. Emerging Trends and Future Perspectives

The next generation of portable sensing systems will move beyond simple analyte detection toward intelligent, connected, sustainable, and application-specific analytical platforms [28,35,36,244,245,246,247,248,249,250]. Future progress will not depend solely on discovering new recognition molecules, improving nanomaterials, or lowering detection limits [251,252,253,254,255,256,257,258,259]. It will depend on the integration of robust recognition chemistry, reliable transducers, automated sample preparation, low-power electronics, secure communication, validated algorithms, scalable manufacturing, and human-centered design [19,38,116,117,118,119,199,200,201,202,251,252,253,254,255,256,257,258,259]. A future portable sensor will be valuable not merely because it detects a biological or chemical target, but because it collects or receives a sample, prepares it appropriately, measures it reliably, interprets the result, communicates useful information, and supports an appropriate decision [19,38,116,117,123,124]. This shift reflects the broader transformation of portable sensing from isolated devices into components of distributed analytical and decision-support systems [110,111,112,113,114,115,260,261,262,263,264].

Realistic expectations must also guide future development. Portable sensors will not eliminate the need for reference laboratories. Instead, they will create layered analytical systems in which field sensors provide rapid screening, monitoring, early warning, and decentralized decision support. In contrast, laboratories provide confirmation, calibration support, traceability, and advanced analysis [163,164,165,166,167,171,175]. The mature role of portable sensing will therefore be complementary rather than substitutive [200,201,218,219,220,221,222]. The most effective systems will combine the immediacy of field sensing with the accuracy and credibility of laboratory methods [175,177,178,193,194,195,196,197].

### 11.1. Integration with Artificial Intelligence

Artificial intelligence will play an increasingly important role in portable sensing because many sensor outputs are complex, noisy, multivariate, and affected by environmental and user-related variation [28,35,36,154,155,156,157,158,159,160,161,162,259]. Machine learning can support interpretation of electrochemical waveforms, optical images, fluorescence patterns, Raman spectra, lateral-flow strips, gas-sensor arrays, wearable signals, and multimodal datasets [13,14,15,16,28,35,36,159,259]. It can also assist in calibration transfer, drift correction, anomaly detection, sensor fusion, interference compensation, image classification, spectral analysis, and automated decision support [151,152,153,159,160,161,162,259]. In sensor arrays, sometimes called electronic noses or electronic tongues, pattern-recognition algorithms can extract useful information from partially selective responses that would be difficult to interpret manually [46,47,159,160,161].

Artificial intelligence and machine learning can enhance portable sensing technologies in six practical ways. First, they improve signal processing by removing noise, correcting baselines, detecting peak features, normalizing images, and extracting useful patterns from weak or unstable signals [28,35,36,154,155,156,157,158,159,259]. Second, they support pattern recognition by classifying lateral flow images, spectral fingerprints, electrochemical profiles, wearable device signals, and sensor array responses into meaningful diagnostic or operational categories [13,14,15,16,28,35,36,159]. Third, they assist calibration transfer by adapting models across instruments, sensor batches, smartphones, temperature, sample matrices, and field environments [151,152,153,160,161]. Fourth, they provide decision support by converting raw signals into positive/negative results, concentration estimates, risk levels, alerts, or recommended actions [28,35,36,116,117,162]. Fifth, they support uncertainty assessment by labeling low-confidence predictions, outliers, invalid tests, drift, and samples outside the calibration range [154,155,156,157,158,162,163,164,165,166,167]. Sixth, they help interpret field data by combining sensor outputs with time, location, environmental conditions, user metadata, and historical measurements [28,30,35,36,159,259].

However, AI should be treated as part of the sensing system rather than as a substitute for strong sensor design [28,35,36,162,259]. A model trained on narrow laboratory data may fail when exposed to real samples, new device batches, degraded reagents, different smartphones, humidity changes, temperature variation, or unexpected interferents [123,151,152,153,175]. Overfitting is a particular risk when datasets are small or collected under overly controlled conditions [154,155,156,157,158,162]. Future AI-enabled sensing systems will need representative datasets, independent validation, uncertainty estimation, explainable outputs, and safeguards against false confidence [158,162,163,164,165,166,167]. In clinical, environmental, food-safety, industrial, and occupational applications, users must know not only what the model predicts but also whether the result is valid within the device’s operating range [116,117,118,163,164,165,166,167]. AI should therefore strengthen measurement reliability, calibration, and decision support, not conceal weak chemistry, poor sampling, or inadequate validation [123,175,259].

### 11.2. Digital Twins and Real-Time Monitoring Networks

Portable sensing will increasingly become part of digital twins and real-time monitoring networks [110,111,112,113,114,115,260,261,262,263,264]. A digital twin is a dynamic model of a physical or biological system that is updated by sensor data and used to support prediction, diagnosis, optimization, or control [260,261,262,263,264]. In healthcare, wearable and point-of-care sensors can feed into patient-specific models for chronic disease monitoring, early warning, medication adjustment, rehabilitation, and remote care [17,18,29,30,263,265,266]. In agriculture, field and greenhouse sensors can support irrigation scheduling, nutrient management, disease forecasting, pest scouting, and climate control [33,262,267,268,269]. In environmental management, distributed portable sensors can map contamination, detect pollution events, and track ecological changes [22,23,24,110,111,112,113,114,115]. In industrial systems, portable and in-line sensors can support predictive maintenance, leak detection, safety monitoring, process optimization, and quality assurance [110,115,260,261,264].

The value of digital twins depends on the quality and continuity of sensor inputs [260,261,262,263,264]. A model is only useful if the data streams are reliable, calibrated, time-stamped, and linked to relevant contextual variables [110,111,112,113,114,115,151,152,153,163,164,165,166,167]. For example, a greenhouse disease model may require not only pathogen detection but also humidity, temperature, leaf wetness, crop stage, and management history [262]. A patient-monitoring model may require biochemical values, activity level, medication use, and physiological signals [263]. A water-quality model may require information on flow, rainfall, sampling locations, and upstream discharge [22,23,24,137,138,139]. Future portable sensing networks will therefore need metadata, calibration records, quality flags, data-fusion methods, and feedback mechanisms that connect measurement to action [110,111,112,113,114,115,260,261,262,263,264].

### 11.3. Multiplexed and Multimodal Detection

Multiplexed and multimodal detection will become increasingly important because many real-world decisions cannot be made based on a single analyte [270,271,272]. Disease diagnosis may require biomarker panels rather than a single marker [270,271,272]. Food quality may depend on microbial load, chemical spoilage indicators, temperature history, packaging atmosphere, and sensory attributes [25,26,27]. Environmental assessment may require nutrients, heavy metals, organic pollutants, pathogens, and physical parameters [22,23,24]. Industrial process control may require simultaneous monitoring of pH, conductivity, dissolved gases, metabolites, solvents, and temperature [110,115]. Wearable health systems may require biochemical, physiological, and behavioral signals to interpret user status accurately [17,18,29,30,104,105,106,107].

Multiplexing allows simultaneous detection of multiple targets, while multimodal sensing combines different transduction mechanisms or data types [270,271,272]. Electrochemical arrays, optical barcodes, multi-analyte lateral-flow tests, microfluidic panels, thermal measurements, molecular panels, and combined optical-electrochemical devices are examples of this trend [32,55,56,57,96,97,98,99,100,101,102,103,270,271,272]. Multimodal systems can improve robustness because one signal may compensate for the weakness of another [28,35,36,95]. They can also provide richer information to support decision-making [116,117,118,272]. However, multiplexing increases complexity. Cross-reactivity, signal overlap, reagent compatibility, calibration burden, data interpretation, and device cost become more difficult [151,152,153,175,272]. Future systems must therefore avoid unnecessary complexity and focus on analyte panels that directly improve decisions [116,117,118]. The best multiplexed device is not the one that measures the most targets, but the one that measures the right targets for a defined use case [116,117,118,272].

### 11.4. Self-Powered and Energy-Harvesting Sensors

Self-powered and energy-harvesting sensors will support long-term, wearable, and remote deployment [213,214,273,274]. Battery capacity, charging requirements, and maintenance burden limit many portable sensing systems [213,214]. Wearable devices may harvest energy from body motion, body heat, sweat chemistry, or flexible photovoltaics [213,214,273,274]. Environmental sensor nodes may use solar cells, microbial fuel cells, vibration energy, chemical gradients, or low-power duty cycling [110,111,112,113,114,115,213]. Industrial and agricultural sensors may combine rechargeable batteries with energy harvesting to reduce maintenance and enable operation in remote locations [110,115,213].

Energy harvesting is attractive, but it introduces variability in available power [213,214,273,274]. A solar-powered environmental node may receive insufficient energy during cloudy periods. A motion-powered wearable may generate different energy levels depending on activity [275,276,277,278]. A biofuel cell-based sensor may depend on the chemical composition of sweat or other environmental fluids [213,214]. Future designs must therefore include ultra-low-power electronics, intermittent sensing, efficient wireless protocols, edge computing, power management, and clear criteria for valid measurement under limited power [213,214,279,280,281]. Self-powered operation should not compromise analytical reliability. A sensor that produces unstable data under variable energy supply may be less useful than a battery-powered device with predictable performance [213,214].

### 11.5. Sustainable and Disposable Sensor Materials

Sustainability will become a more important design criterion as portable sensing expands into large-scale testing [275,276,277,278]. Many portable sensors are designed for single use because disposability reduces contamination, simplifies operation, avoids surface regeneration, and supports calibration control [11,12,19,96,97]. However, high-volume use of disposable plastics, metals, batteries, electronic components, biological reagents, and nanomaterials can create environmental burdens [275,276,277,278]. Public health testing, food-safety screening, agricultural monitoring, and environmental surveillance may generate large quantities of sensor waste if sustainability is not considered early [11,12,25,26,27,96,275,276,277,278].

Future research should explore biodegradable substrates, paper-based materials, recyclable electronics, low-toxicity nanomaterials, reduced reagent volumes, modular readers, replaceable cartridges, and waste-minimizing packaging [96,97,98,275,276,277,278]. Life-cycle assessment should be incorporated into device development, especially for high-volume applications [277,278]. Sustainable design must be balanced with analytical performance, biosafety, shelf life, cost, and user convenience [19,38,183,184,185]. A biodegradable sensor that cannot preserve reagent stability or prevent contamination will not be practical [140,141,142,143,183,184,185].

Conversely, a highly accurate disposable device may be difficult to justify if it creates excessive waste [275,276,277,278]. The future challenge is to design portable sensing systems that are analytically credible, economically viable, and environmentally responsible [275,276,277,278].

### 11.6. Toward Fully Integrated Sample-to-Answer Systems

Fully integrated sample-to-answer systems remain a central goal of portable sensing [19,124,128]. The ideal system would accept a raw or minimally prepared sample, perform sample preparation, reagent delivery, reaction, detection, quality control, interpretation, data storage, communication, and decision support without specialist intervention [19,38,123,124,128]. Such systems may use closed cartridges, automated or passive microfluidics, dried reagents, embedded calibration, internal controls, portable readers, mobile interfaces, cloud-linked records, and secure data management [19,38,124,128,168,169,170]. Integration is especially important for nucleic acid testing, immunoassays, pathogen detection, food-safety screening, and environmental monitoring, where manual sample preparation often dominates complexity [20,21,55,56,57,123,124,125,126,127,128,217].

Achieving sample-to-answer operation requires more than engineering convenience. It is essential for reducing user error, contamination, variability, and training burden [116,117,118,123,124]. However, full integration also increases design complexity, manufacturing cost, and validation requirements [19,38,175]. Closed cartridges must manage sample loading, fluid movement, reagent storage, reaction timing, waste containment, and sensor alignment [19,38,124,128]. They must also remain stable during storage and shipping [183,184,185]. Future progress will depend on simplifying these systems while maintaining analytical reliability [19,38,123,124,128]. The practical endpoint of portable sensing is not the smallest sensor, but the most dependable workflow [19,38,116,117,118].

### 11.7. Edge Computing and Local Decision Support

Edge computing will become increasingly important as portable sensors generate more data [279,280,281]. Instead of sending all raw data to the cloud, devices can process signals locally, reduce communication demands, protect privacy, and provide immediate feedback [279,280,281]. Local processing is especially useful when connectivity is unreliable, when rapid alarms are required, or when data are sensitive [168,169,170,279,280,281]. Wearable health sensors, occupational safety monitors, environmental nodes, and industrial devices may all benefit from edge computing [29,30,104,105,106,107,110,111,112,113,114,115].

Edge processing can support signal filtering, baseline correction, calibration checks, anomaly detection, preliminary classification, and data compression [144,145,146,147,279,280,281]. Cloud systems can then receive summarized results, calibration updates, quality-control records, and selected raw data when needed [110,111,112,113,114,115,116,117,279,280,281]. This hybrid architecture can improve responsiveness and reduce dependence on continuous connectivity [279,280,281]. However, edge algorithms must be validated and controlled, as local software decisions may directly influence user actions [162,231,232,233]. Firmware updates, model versioning, cybersecurity, and auditability will become important components of future portable sensing products [168,169,170,231,232,233].

### 11.8. Practical Design Recommendations for Future Research

Future research on portable sensing should begin with the use case rather than the material [116,117,118,119,200,201]. Researchers should define the target analyte, target sample, user group, operating environment, decision threshold, acceptable error rate, required time-to-result, cost target, storage condition, and reference method before selecting the recognition chemistry and transducer [116,117,118,119,171,175]. This approach does not restrict scientific creativity. Instead, it directs creativity toward systems that address real needs [116,117,118,119,200,201]. It also helps identify whether the main barrier is recognition, sample preparation, transduction, calibration, packaging, data processing, manufacturing, or adoption [19,38,123,175].

Future publications should report complete workflows [175,193,194,195,196,197]. They should describe how the sample is collected, how much sample is required, whether dilution, extraction, lysis, filtration, incubation, or washing is needed, how reagents are stored, how many user steps are required, how long the full test takes, how results are interpreted, and how waste is handled [123,175,193,194,195,196,197]. Reporting only the sensing step gives an incomplete picture because field users experience the entire workflow [19,38,116,117,118]. A sensor with excellent electrode response may still be impractical if the sampling and preparation steps are unrealistic [123,175].

Validation in realistic matrices should occur early [123,175]. Buffer studies are useful for understanding mechanisms, but they should be followed by spiked real matrices and then unspiked real samples [123,175]. Matrix effects should be quantified rather than treated as secondary complications [22,23,24,25,26,27,123]. Recovery, bias, precision, interference, false-positive rates, false-negative rates, and failure modes should be reported under conditions resembling intended use [175,176,177,178,179,180,181,182]. This practice would make academic results more comparable and more useful for industrial translation [175,193,194,195,196,197].

Manufacturing thinking should also be included in prototype design [203,204,205,206,207,229,230]. Researchers should ask whether the fabrication method can be scaled, whether material suppliers are reliable, whether batch variation can be controlled, whether the device can be packaged, and whether quality-control tests can be performed [175,179,180,181,182,183,184,185,203,204,205,206,207]. A sensor that can be printed reproducibly and stored reliably may have greater practical impact than a complex hand-fabricated device with slightly better sensitivity [203,204,205,206,207]. Design-for-manufacture should be viewed as part of translational research, not as a later commercial detail [119,203,204,205,206,207,229,230].

Future devices should also be designed to detect failures [116,117,175]. Portable sensors used by non-specialists should identify insufficient sample volume, invalid flow, expired reagents, abnormal temperature, low battery, calibration failure, out-of-range signals, poor optical images, and communication errors [116,117,118,175]. Internal controls, reference channels, electronic self-checks, software flags, and clear error messages can reduce incorrect interpretation [116,117,118,175]. A device that fails safely is more trustworthy than a device that produces a numerical result under all conditions [116,117,175].

**Figure 6:**
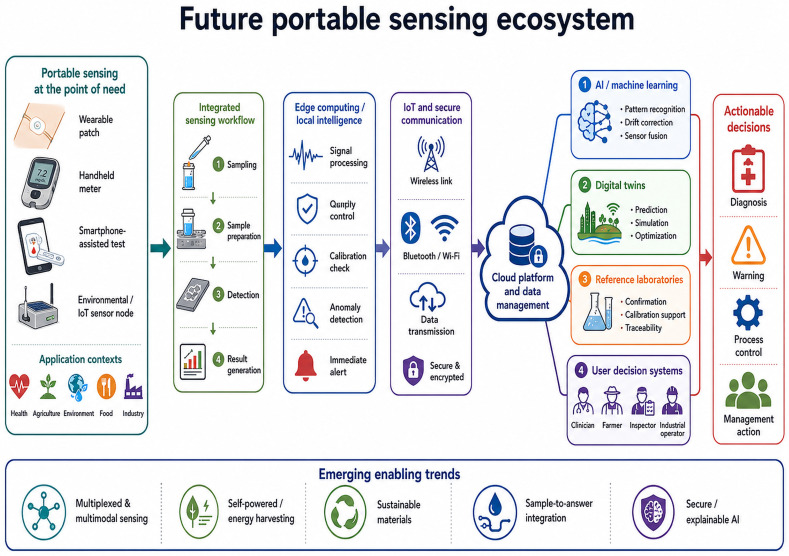
Future portable sensing ecosystem.

The future portable sensing ecosystem is shown in Figure 6. This figure shows portable sensors connected to AI, digital twins, edge computing, IoT networks, reference laboratories, cloud platforms, and user decision systems.

### 11.9. Interdisciplinary Collaboration and Standardized Reporting

Interdisciplinary collaboration will be essential for the next stage of portable sensing [116,117,118,119,200,201]. Chemists, biologists, electrical engineers, mechanical engineers, data scientists, clinicians, agronomists, food scientists, environmental specialists, industrial users, manufacturers, and regulators each recognize different risks [116,117,118,119,200,201]. Collaboration only at the end of development is often too late. Early collaboration can prevent unrealistic sample preparation, unmanufacturable materials, unusable interfaces, poor data architecture, inadequate validation plans, and regulatory obstacles [116,117,118,119,200,201].

Standardized reporting guidelines would also accelerate progress [193,194,195,196,197]. Such guidelines could include device description, sample type, sample preparation, number of devices, batch information, calibration method, environmental conditions, real-sample validation, reference method comparison, stability testing, user steps, total assay time, estimated cost, data-processing method, and failure modes [175,193,194,195,196,197]. Standardized reporting would improve reproducibility, support meta-analysis, help industry identify promising technologies, and clarify which prototypes are ready for translation [193,194,195,196,197]. The field will progress most effectively when innovation in sensing chemistry is combined with rigorous engineering, realistic field testing, transparent reporting, and clear understanding of user needs [116,117,118,119,175,193,194,195,196,197].

### 11.10. Future Outlook

The future of portable sensing will be shaped by integration, realism, and decision relevance [19,38,116,117,118,119]. More data will not automatically produce better decisions, and more complex devices will not always be more useful [116,117,118,158,162]. Practical value depends on matching the analytical method to the decision problem [116,117,118,171,175]. In decentralized healthcare, integrated portable systems can expand access to diagnostics and monitoring, but they must be connected to care pathways [200,201,218,219,220,221,222,265,266,267,268,269]. In agriculture, they can support earlier detection of plant disease, better nutrient management, and more precise use of pesticides and fertilizers, but they must be affordable and actionable [33,262,267,268,269]. In environmental surveillance, they can provide spatial and temporal data that are impossible to collect through laboratory sampling alone, but they must be maintained in calibration and data quality [22,23,24,110,111,112,113,114,115]. In food safety, they can support rapid screening across supply chains, but positive results may still require confirmation [25,26,27]. In industrial systems, they can reduce downtime and improve process control, but they must be rugged, maintainable, and trusted [110,115,260,261].

Current constraints show where future research should be directed. Portable systems still face incomplete sample preparation, matrix interference, reagent instability, sensor drift, weak calibration transfer, limited battery life, device-to-device variation, poor long-term field validation, cybersecurity risks, and uncertain regulatory pathways [19,30,38,39,40,49,50,116,117,118,119]. Therefore, future development should emphasize closed sample-to-answer cartridges, antifouling and self-cleaning sensor surfaces, dry and room-temperature-stable reagents, built-in quality-control materials, automatic calibration and drift correction, low-power electronics, standardized smartphone or reader interfaces, explainable AI models, and validation with real users and real samples [13,14,15,16,19,28,35,36,116,117,118,119,151,152,153,154,155,156,157,158,159,160,161,162]. More attention is also needed on scalable manufacturing, sustainable disposable materials, maintenance procedures, interoperability with health, farm, environmental, and industrial databases, and cost models suitable for small clinics, farms, and field agencies [110,111,112,113,114,115,275,276,277,278,279,280,281].

The broad direction is clear: portable sensing is becoming part of larger information networks rather than remaining a collection of isolated devices [110,111,112,113,114,115,260,261,262,263,264]. The strongest future systems will combine robust chemistry, reliable hardware, realistic sampling, validated algorithms, secure data management, sustainable materials, and user-centered deployment [19,38,116,117,118,119,275,276,277,278,279,280,281,282,283]. When these elements are developed together, portable sensing systems can become dependable tools for biological and chemical analyses in healthcare, agriculture, environmental protection, food safety, industry, public safety, and personal monitoring [200,201,202,265,266,268,269,282,284,285,286,287,288,289,290,291,292].

## 12. Conclusions

Portable sensing systems represent a significant transformation in the fields of biological and chemical analyses. They shift analytical information from centralized laboratories to decision-making locations, including clinics, homes, farms, greenhouses, food processing plants, rivers, factories, emergency sites, workplaces, and personal environments. Advances in fields such as identification chemistry, nanomaterials, microfluidics, flexible electronics, microsensors, smartphones, wireless communications, and data analytics have facilitated this shift. The value of portable sensing systems is particularly evident when decision-making time is tight, sampling points are widely distributed, laboratory access is limited, or repetitive monitoring is more useful than a single centralized measurement. In these situations, portable sensing systems can provide rapid, convenient, and actionable information where needed.

However, portable sensing systems should not be considered a replacement for traditional laboratory instruments. Laboratory methods remain essential for reference analysis, validation, regulatory enforcement, method development, calibration support, and high-resolution characterization. The maturation of portable sensing systems complements these methods. Portable systems enable screening, monitoring, guiding immediate action, and identifying suspicious samples, while laboratories provide traceability, confirmatory evidence, and advanced analytical capabilities. Therefore, the future analytical ecosystem will combine distributed sensing with centralized expertise, rather than replacing one with the other.

The most important conclusion of this review is that portable sensing must be evaluated as a complete system. Identification elements and sensors are important, but they are only one part of the final device. Sample acquisition, sample preparation, reagent storage, calibration, power supply, packaging, user interface, data processing, communications, cybersecurity, validation, manufacturing, and regulatory approval are equally important. Many promising prototypes fail because they address only one analytical problem rather than the entire workflow. Even if a sensor performs well in laboratory buffers, it may fail in field use if it cannot withstand complex matrices, calibration cannot be transferred across batches, reagents degrade during storage, biocontamination and drift are uncontrolled, the user interface is error-prone, or results cannot be correlated with practical decisions.

Future progress will depend on fully integrated sample-to-result systems. Such systems will integrate robust sensing chemistry, reliable hardware, miniaturized sample handling, automated quality control, intelligent data interpretation, secure communications, and user-centric deployment. Artificial intelligence, digital twins, multiplexing and multimodal detection, self-powered electronics, sustainable single-use materials, edge computing, and IoT networks will drive this development, but only if they are validated under real-world conditions. More data, higher complexity, or lower detection limits do not necessarily lead to better decision-making. The key question is whether the system can provide sufficiently accurate information at the right time, in the right place, and at an acceptable cost to support appropriate action.

Artificial intelligence and machine learning will further strengthen portable sensing by improving signal processing, pattern recognition, calibration transfer, decision support, uncertainty assessment, and field data interpretation. They can remove noise, correct baselines, classify lateral-flow images, interpret spectra and electrochemical profiles, adapt calibration models across devices and sample matrices, and convert raw signals into risk levels, alerts, or recommended actions. They can also identify invalid tests, low-confidence predictions, drift, outliers, and samples outside the calibration range. By combining sensor outputs with time, location, environmental conditions, and historical data, AI can make portable sensing more reliable and decision-oriented.

In the short term, practical improvement should focus on problems that can be solved within existing device architectures. These include better sample pretreatment, improved antifouling surfaces, more stable dry reagents, internal standards, reference zones, automatic background correction, calibration-transfer models, drift alarms, clearer user instructions, smartphone holders, and simple quality-control indicators [13,14,15,16,19,30,38,39,40,49,50,116,117,118,119,151,152,153,154,155,156,157,158,159,160,161,162]. Such improvements can increase reliability without waiting for entirely new sensing platforms. In the long term, the goal should be solution-oriented systems: fully integrated, low-cost, robust, validated, and field-usable handheld sensor networks that combine sampling, preparation, detection, interpretation, secure communication, cloud or edge analytics, maintenance planning, and decision support [110,111,112,113,114,115,260,261,262,263,264,275,276,277,278,279,280,281]. These systems should be validated across real users, real samples, device batches, climates, and application sites [116,117,118,119,163,164,165,166,167].

Therefore, the field should move towards standardized reporting, comparative reference methods, real-world application matrix validation, field testing, usability studies, manufacturability assessments, sustainability assessments, and early collaboration among scientists, engineers, clinicians, agricultural experts, environmental experts, industrial users, manufacturers, regulatory agencies, and end-users. Through this broader, system-level approach, portable sensing systems can become reliable tools for decentralized healthcare, precision agriculture, environmental monitoring, food safety management, public health protection, industrial process control, forensic analysis, occupational safety, and personal monitoring.

## Figures and Tables

**Table 1:** Comparison between laboratory-based analysis and portable sensing systems.

Comparison Item	Laboratory-Based Analysis	Portable Sensing Systems	Main Implication for Use	Literature
Analytical accuracy	Usually provides high sensitivity, selectivity, reproducibility, and quantitative reliability under controlled conditions. Laboratory instruments can often achieve lower limits of detection, wider dynamic ranges, and stronger discrimination between closely related analytes.	Usually provides sufficient accuracy for screening, triage, monitoring, or process guidance, but may have higher LOD, narrower linear range, and greater device-to-device variation.	Laboratory analysis remains preferred for confirmatory testing, regulatory enforcement, and complex quantification, whereas portable sensing is useful when timely information is more important than maximum analytical precision.	[30,31,37,38,39,40]
Limit of detection and linearity	LOD and linearity are commonly established using validated calibration standards, controlled sample preparation, stable instruments, and traceable reference methods. The linear range can be optimized by dilution, extraction, and instrument settings.	LOD and linearity may be affected by miniaturized reaction volume, weak signal intensity, matrix interference, reagent stability, optical background, electrode fouling, and limited calibration transfer.	Portable devices should report LOD and linearity not only in buffer or standard solution, but also in real biological, food, environmental, or industrial samples.	[30,31,37,38,39,40]
Speed of result and response time	Results may be delayed by sample collection, transportation, pretreatment, instrument scheduling, data review, and reporting. The actual analytical response may be fast, but the overall turnaround time can be long.	Provides rapid or real-time results at or near the sampling site. Response time is often a major advantage of portable systems, especially for point-of-care, food safety, environmental, and industrial decisions.	Portable sensing is valuable when immediate intervention, screening, or repeated monitoring is more important than centralized high-precision analysis.	[33,34,38,39,40]
Infrastructure	Requires controlled laboratory space, capital-intensive instruments, stable power supply, trained operators, maintenance, calibration materials, sample logistics, and waste management.	Requires limited infrastructure and can often operate in clinics, farms, food-processing sites, environmental fields, production lines, homes, or wearable settings.	Portable systems decentralize analysis and expand access beyond specialized laboratories, but they must be robust against field temperature, humidity, vibration, dust, and user variation.	[29,30,31,33,34,37,38]
User skill	Usually operated by trained technicians or analysts who understand sample preparation, instrument operation, calibration, quality control, and data interpretation.	Designed for simpler operation by clinicians, patients, farmers, inspectors, workers, or consumers. However, correct sampling, timing, calibration, and interpretation are still essential.	User training shifts from complex instrument operation to reliable field procedures, correct sample handling, and decision interpretation.	[38,39,40]
Actual sample and matrix effect	Laboratory methods often include standardized pretreatment steps such as filtration, dilution, extraction, digestion, separation, or purification to reduce matrix interference.	Portable systems must often analyze whole blood, sweat, saliva, urine, plant sap, food homogenate, soil extract, wastewater, air samples, or industrial fluids with minimal pretreatment.	Performance should be evaluated in actual samples, not only in clean buffer. Matrix effect, fouling, turbidity, viscosity, pigments, salts, proteins, and interferents must be considered.	[29,30,31,33,34,37,38,39,40]
Recovery and accuracy in real samples	Recovery studies are commonly performed using spiked samples, reference materials, or comparison with standard laboratory methods. This supports method validation and quantitative reliability.	Recovery may vary with sample type, user handling, reagent release, incomplete extraction, evaporation, temperature, humidity, or signal drift. Field recovery data are often weaker than laboratory recovery data.	Portable sensors should report recovery in relevant real samples and, whenever possible, compare results with accepted reference methods.	[30,31,37,38,39,40]
Cost structure	High capital cost and recurring costs for maintenance, reagents, technical labor, quality control, infrastructure, and sample transport.	Lower cost per test and suitable for repeated, distributed, or high-frequency measurements. Disposable strips, paper devices, smartphone readers, and wearable platforms can reduce access barriers.	Portable systems support frequent screening and monitoring when laboratory testing is impractical, too slow, or too costly.	[29,30,33,34,38]
Calibration and drift	Strong calibration traceability and quality control can be maintained under controlled conditions. Instruments can be recalibrated regularly using certified standards.	More affected by sensor drift, device variability, reagent degradation, environmental effects, matrix interference, user handling, and calibration-transfer limitations.	Field data require calibration management, internal standards, drift correction, quality-control checks, and sometimes laboratory confirmation.	[30,39,40,49,50]
Stability and storage	Reagents, standards, and instruments are usually stored under controlled laboratory conditions, including controlled temperature, humidity, light exposure, and maintenance schedules.	Portable devices may require room-temperature storage, long shelf life, dry reagent formats, stable biological recognition elements, rugged packaging, and resistance to heat, humidity, vibration, and transport stress.	Stability testing should include reagent stability, sensor shelf life, operational stability, repeated-use stability, and storage under realistic field conditions.	[29,30,31,33,34,37,38,39,40]
Traceability and regulatory role	Provides traceable data suitable for confirmatory testing, enforcement, clinical diagnosis, advanced research, and regulatory documentation.	Often more suitable for screening, triage, process guidance, preliminary decisions, field monitoring, and decentralized decision support.	Portable sensing should complement rather than fully replace reference laboratories, especially when regulatory or clinical confirmation is required.	[31,37,38,39,40]
Validation and quality control	Validation usually includes accuracy, precision, LOD, LOQ, linearity, selectivity, robustness, recovery, reproducibility, uncertainty, and comparison with reference methods.	Validation must additionally consider user operation, sample-to-answer workflow, environmental tolerance, calibration transfer, connectivity, cybersecurity, manufacturability, and real-world usability.	Portable sensing requires system-level validation, not only sensor-element validation. The complete device, sample workflow, data processing, and decision output must be tested.	[30,31,37,38,39,40]
Decision proximity	Analytical results are often separated from the point of need by time and location. The result may arrive after the ideal decision window has passed.	Measurement occurs close to the sample, user, patient, process, animal, crop, food product, or field condition.	The main advantage is decision proximity: rapid action can be executed.	

**Table 2:** Comparison of signal transduction technologies for portable sensing.

Transduction Technology	Signal Principle	Main Advantages for Portable Sensing	Key Limitations in Field Use	Representative Applications	Literature
Electrochemical sensors	Convert recognition events into current, potential, impedance, conductance, or charge-transfer signals.	Highly suitable for miniaturization, low power demand, low-cost fabrication, disposable strips, wearable patches, handheld meters, and direct electronic readout.	Electrode fouling, reference-electrode instability, temperature effects, drift, nonspecific adsorption, redox interferents, and matrix effects may reduce reliability.	Glucose and lactate meters, pH and ion detection, immunosensors, nucleic acid sensors, heavy-metal and pesticide detection, and biofilm monitoring.	[22,29,49,50,51,52,53,54,74,75,76,77,78,79]
Optical sensors	Convert recognition events into color, absorbance, fluorescence, chemiluminescence, plasmonic, Raman, or image-based signals.	Simple visual readout is possible; compact readers, LEDs, photodiodes, fiber optics, and smartphone cameras enable low-cost field use and digital recording.	Ambient light, turbidity, background color, camera variation, optical path length, angle, distance, user perception, and reagent stability affect quantification.	Paper devices, lateral-flow assays, dipsticks, test strips, fluorescence assays, smartphone assays, plasmonic sensing, and Raman/SERS detection.	[7,11,12,13,14,15,16,45,46,55,56,57,80,81,82,83,84,85]
Mass-sensitive sensors	Detect target binding through mass change, resonance shift, surface stress, or acoustic response.	Provide label-free and direct detection of binding events, useful when labels are undesirable.	Require stable oscillators, surface functionalization, vibration control, humidity management, fluidics, environmental compensation, and fouling control.	Biomolecular interaction studies, gas sensing, pathogen detection, protein binding, environmental monitoring, and vapor detection.	[86,87,88,89]
Thermal and calorimetric sensors	Measure heat generated or absorbed by reactions, binding, metabolism, gas sorption, or catalytic conversion.	Can operate without optical transparency or electroactive species; useful for reactions with strong thermal signatures.	Small heat signals are easily affected by heat loss, temperature fluctuation, self-heating, poor insulation, and baseline instability.	Enzyme reactions, immunoreactions, microbial activity, fermentation monitoring, gas sorption, and catalytic processes.	[5,6,90]
Electrical and field-effect transistor sensors	Detect changes in surface charge, gate potential, conductivity, electric field, or carrier concentration near a sensing channel.	Enable label-free detection, miniaturization, direct electronic readout, and possible integration with semiconductor fabrication and circuits.	Debye screening, unstable surface chemistry, device variability, threshold drift, nonspecific adsorption, packaging, and fluidic integration remain major barriers.	Ion, gas, protein, nucleic acid, cell, and small-molecule sensing using ISFETs, nanowires, graphene, CNTs, and organic electrochemical transistors.	[29,30,91,92,93,94,95]

**Table 3:** Portable platform designs and their operational characteristics.

Platform Design	Operational Characteristics	Main Advantages	Main Limitations	Suitable Uses	Literature
Paper-based analytical devices	Use porous substrates to move samples by capillary action; include lateral-flow assays, dipsticks, colorimetric strips, paper microfluidics, and electrochemical paper sensors.	Low cost, disposable, lightweight, simple, pump-free, small sample volume, and suitable for mass production.	Flow variability, humidity effects, evaporation, reagent instability, uneven color, limited sample preparation, and subjective visual reading.	Rapid screening, low-resource testing, environmental contaminants, pathogens, pesticides, ions, and metabolites.	[11,12,13,14,15,16,24,55,56,57,82,96,97,98]
Lab-on-a-chip and microfluidic devices	Manipulate small fluid volumes through channels, chambers, valves, pumps, droplets, membranes, mixers, and detection zones.	Integrate sample preparation and detection; reduce reagent use; improve reaction kinetics; enable multiplexing and cleanup.	Clogging, bubbles, evaporation, leakage, sealing problems, reagent storage, waste containment, and difficult field operation.	Nucleic acid testing, immunoassays, cell analysis, pathogen detection, sweat collection, preconcentration, and sample cleanup.	[4,10,19,32,33,99,100,101,102]
Smartphone-based sensing systems	Use cameras, processors, storage, wireless communication, GPS, displays, and apps as sensing interfaces.	Widely accessible; supports imaging, computation, geotagging, cloud connection, user guidance, and remote consultation.	Phone-model variation, lighting, distance, angle, software updates, cybersecurity, privacy, and validation challenges.	Colorimetric strips, lateral-flow assays, fluorescence readers, microfluidic devices, microscopes, spectrometers, and electrochemical modules.	[13,14,15,16,43,82,83,103]
Wearable and flexible sensors	Attach to skin, clothing, animals, plants, packages, or flexible surfaces for repeated or continuous monitoring.	Provide proximity over time, trend data, exposure history, flexibility, wireless operation, and integration with physical sensors.	Motion artifacts, sweat-rate variability, skin irritation, biofouling, drift, adhesive failure, power demand, and interpretation uncertainty.	Sweat, saliva, breath, interstitial fluid, exposure monitoring, fatigue, hydration, gases, pesticides, and localized surface sensing.	[17,18,29,30,104,105,106,107]
Handheld and field-deployable instruments	Dedicated portable readers or analyzers with controlled measurement conditions and battery-powered operation.	Better quantification, rugged housing, controlled optics/electronics, calibration, data storage, traceability, and quality checks.	Higher cost, training, maintenance, calibration, consumables, software updates, and service requirements.	Field inspection, food safety, environmental testing, PCR, Raman/NIR, gas detection, ATP meters, and immunoassay readers.	[19,22,23,24,25,26,27,45,74,75,108,109,110,111]
Wireless sensor networks and IoT platforms	Distributed nodes collect, transmit, store, visualize, and analyze data across space and time.	Reveal trends, gradients, hotspots, abnormal events, and enable alarms, dashboards, remote access, and automated control.	Drift, calibration mismatch, data gaps, battery depletion, communication failure, cybersecurity, interoperability, and maintenance burden.	Farms, greenhouses, rivers, factories, hospitals, cold chains, livestock facilities, urban environments, and occupational safety.	[17,18,22,23,24,29,30,104,105,106,107,108,109,110,111,112,113,114,115,116,117,118]
User-centered workflow and packaging	Integrate sample handling, reagent protection, waste containment, software, hardware, packaging, and human factors.	Improves usability, reliability, storage, transport, interpretation, and decision relevance.	Poor workflow or packaging can lead to user error, leakage, contamination, reagent degradation, calibration failures, or user rejection.	Commercial products and field systems requiring robust, affordable, interpretable, and decision-oriented operation.	[3,19,38,116,117,118,119,120,121,122]

**Table 4:** Sample types, matrix challenges, and preparation strategies in portable analysis.

Sample Type	Representative Matrices	Main Matrix Challenges	Portable Preparation Strategies	Design Implication	Literature
Biological fluids	Whole blood, serum, plasma, urine, saliva, sweat, tears, breath condensate, interstitial fluid, wound fluid	Cells, proteins, salts, enzymes, clotting factors, lipids, redox interferents, pH variation, viscosity, evaporation, and local physiological variation	Filtration, dilution, plasma separation, reagent mixing, membrane separation, enzymatic pretreatment, immunocapture, and closed cartridges	Biological analysis requires matrix-specific preparation because performance in buffer may not predict performance in real samples.	[29,30,104,105,106,107,123,125,126,127,128,129,130,131,132,133,134]
Plant, animal, and microbial samples	Plant sap, tissue extracts, animal fluids, milk, microbial cultures, wound fluid	Pigments, phenolics, sugars, organic acids, fibers, particulates, proteins, fats, pathogens, drug residues, enzymes, debris, and microbial heterogeneity	Clarification, washing, concentration, lysis, extraction, magnetic capture, nucleic-acid extraction, and cartridge containment	Target release and interference removal must be integrated with detection, especially for nucleic acid, protein, toxin, and pathogen assays.	[22,23,25,26,27,123,125,126,127,128,129,130,131]
Water and environmental samples	Drinking water, river water, seawater, wastewater, soil extracts, air, aerosols, surface swabs	Suspended solids, organic matter, microorganisms, humic substances, salinity, competing ions, chlorine, metals, variable pH, humidity, and flow variation	Filtration, sedimentation, dilution, preservation, solid-phase extraction, preconcentration, pH adjustment, and controlled air sampling	Field preparation must reduce matrix effects while remaining simple enough for non-laboratory users.	[22,23,24,123,135,136,137,138,139]
Food samples	Milk, meat, fruit juice, grains, vegetables, oils, processed foods	Fats, proteins, carbohydrates, pigments, spices, salts, preservatives, fibers, residues, and heterogeneous texture	Homogenization, extraction, clarification, dilution, filtration, cleanup, and selective capture	Food sensing often requires sample-specific extraction or cleanup before reliable portable detection is possible.	[25,26,27,123,135,136]
Industrial and process samples	Effluents, process fluids, workplace gases, pesticide formulations, equipment residues	Solvents, surfactants, oils, corrosive compounds, emulsions, high ionic strength, particles, and unknown interferents	Dilution, filtration, preservation, separation, preconcentration, compatible cartridges, and protective packaging	Sensors must be protected from chemically aggressive matrices and calibrated for real process conditions.	[22,23,24,123,138,139]
Reagent-dependent assays	Enzyme, antibody, primer, nanoparticle, buffer, redox mediator, fluorescent probe, or extraction reagent systems	Leakage, evaporation, freezing, contamination, short shelf life, poor rehydration, humidity, heat, oxygen, and mechanical damage	Lyophilization, dried reagents, sealed cartridges, foil pouches, desiccants, stabilizers, polymers, sugars, protein protectants, and temperature indicators	Reagent stability may determine field success as much as analytical sensitivity.	[3,5,6,7,8,9,19,20,21,38,53,54,140,141,142,143]

**Table 5:** Technical limitations and engineering solutions in portable sensing.

Technical Limitation	Main Cause or Challenge	Engineering Solutions	Design Implication	Literature
Sensitivity versus portability	High sensitivity may require complex optics, low-noise electronics, amplification, enrichment, heating, washing, or long incubation, increasing size, cost, power demand, and user burden.	Define decision-relevant detection limits; use enrichment only when needed; optimize robustness, matrix tolerance, reproducibility, and ease of use.	Sufficient sensitivity for the intended decision is often more valuable than laboratory-level detection limits.	[3,19,22,23,24,25,26,27,33,38,45,59,60,61,62,116,117,124,171,173,174,175,199,200,201,202]
Reproducibility of fabrication	Manual modification, drop casting, small-batch nanomaterials, hand-cut structures, and individually optimized conditions lead to device-to-device and batch-to-batch variation.	Use scalable fabrication, standardized surface chemistry, automated reagent deposition, in-process quality control, lot testing, and tolerance analysis.	Manufacturability should be considered early, not after the sensing concept is optimized.	[19,38,53,54,77,79,96,97,98,117,119,151,152,153,175,179,180,181,182,183,184,185,203,204,205,206,207,208]
Biofouling and sensor drift	Proteins, cells, particles, fats, salts, organic matter, skin debris, microbial growth, reagent degradation, electrode instability, and temperature variation alter signals over time.	Apply antifouling coatings, protective membranes, disposable elements, reference channels, periodic calibration, surface regeneration, signal normalization, and drift-correction algorithms.	Long-term and repeated-use devices must be validated in real matrices over realistic operating times.	[22,23,24,25,26,27,29,30,49,50,53,54,76,104,105,106,107,123,125,126,127,132,133,134,135,136,137,138,139,140,141,142,143,151,152,153,160,161,199,209,210,211,212]
Power supply and durability	Pumps, heaters, optics, wireless communication, displays, processors, and amplification increase energy demand; field use exposes devices to dust, water, impact, vibration, heat, and chemicals.	Use low-power electronics, batteries, energy harvesting, rugged housings, sealed connectors, chemical-resistant materials, flexible designs, and environmental durability testing.	Power and durability are core design requirements, not final packaging details.	[13,14,15,16,19,20,21,29,30,38,59,60,61,62,104,105,106,107,110,115,183,184,185,213,214,215,216]
Incomplete workflow integration	Small sensors may still require pipettes, centrifuges, microscopes, incubators, refrigerated reagents, washing steps, or expert interpretation.	Develop sample-to-answer cartridges, sealed reagents, passive or automated fluidics, waste containment, built-in calibration, and quality checks.	True portability requires that the entire workflow, not just the sensor, function outside the laboratory.	[19,20,21,32,38,55,56,57,116,117,123,124,125,126,127,128,200,201,202,217]
Cost, manufacturability, and adoption	Expensive materials, cartridges, proprietary readers, maintenance, training, invalid tests, data handling, and confirmatory testing increase total cost.	Evaluate cost per useful decision; simplify design; align with user workflows, supply chains, price constraints, and maintenance capacity.	Practical value depends on affordability, usability, and adoption, not academic novelty alone.	[3,7,8,19,38,116,117,118,119,200,201,218,219,220,221,222]
Standardization and regulatory acceptance	Different domains require different validation, documentation, traceability, reference methods, quality systems, and risk evidence.	Report matrix, batches, operators, environment, calibration, reference method, failure rate, stability, and intended-use limits.	Transparent validation improves comparability, reproducibility, regulation, and adoption.	[25,26,27,163,164,165,166,167,171,172,173,174,175,176,177,178,179,180,181,182,183,184,185,186,187,188,189,190,191,192,193,194,195,196,197,198,223,224,225,226]

**Table 6:** Gap between academic prototype and industrial product.

Translation Issue	Academic Prototype Emphasis	Industrial Product Requirement	Practical Solution	Literature
Evaluation criteria	Novel materials, low detection limits, selectivity, rapid response, and proof-of-concept detection under controlled conditions.	Reliable, timely, affordable, interpretable, and actionable results under intended-use conditions.	Shift from sensor-centered research to system-centered development with defined intended use.	[3,4,19,38,116,117,118,119,171,175,199,200,201,202,225,226,227,228]
Field readiness	Testing in clean buffers, controlled temperature, trained operation, and freshly prepared devices.	Tolerance to real samples, humidity, dust, vibration, sunlight, storage, rough handling, and limited user training.	Include rugged packaging, protected reagents, simple sample introduction, clear outputs, and quality checks.	[19,38,116,117,118,123,171,175,183,184,185,186,187,188,189,200]
Practical reliability	Low detection limit from few devices, spiked samples, or optimized conditions.	Reproducibility across samples, lots, operators, environments, and storage periods, with known false results and failure rates.	Validate with real matrices, blind samples, controls, confidence intervals, deployment protocols, and confirmatory pathways.	[20,21,47,123,124,125,126,127,128,159,160,161,171,172,173,174,175,176,177,178,179,180,181,182,193,194,195,196,197,199,200,201,202,217]
Sample preparation	External centrifugation, extraction, filtration, washing, pipetting, incubation, or reagent mixing may be acceptable.	Sample-to-answer operation with minimal handling, contamination control, and integrated preparation.	Use cartridges, closed fluidics, integrated lysis, extraction, washing, amplification, filtration, waste containment, and reagent storage.	[19,20,21,24,25,26,27,38,55,56,57,59,60,61,62,123,124,125,126,127,128,135,136,200,217]
Manufacturing reproducibility	Hand-prepared films, drop casting, manual functionalization, small-batch materials, and best-case results.	Controlled materials, specifications, acceptance criteria, lot release, quality assurance, and calibration consistency.	Apply design-for-manufacture, automated dispensing, screen printing, roll-to-roll processing, injection molding, laser patterning, and batch testing.	[53,54,63,64,65,77,79,96,97,98,119,151,152,153,171,175,179,180,181,182,183,184,185,203,204,205,206,207,229,230]
Stability and shelf life	Devices may be tested soon after fabrication under favorable storage conditions.	Predict shelf life during shipping, storage, temperature cycling, humidity, and field use.	Use desiccants, oxygen barriers, foil pouches, stabilizers, lyophilization, sealed cartridges, thermal protection, and real-time stability testing.	[3,19,38,53,54,140,141,142,143,183,184,185,200,201,225]
Calibration and inter-device consistency	Calibration may be device-specific or limited to laboratory conditions.	Transferable calibration across devices, lots, users, environments, smartphones, wearables, and sensor networks.	Use internal references, onboard controls, ratiometric signals, environmental compensation, standardized manufacturing, and field verification.	[13,14,15,16,80,81,82,104,105,106,107,108,109,110,111,112,113,114,115,132,133,134,151,152,153,159,160,161,162,163,164,165,166,167,175,179,180,181,182,183,184,185]
User-centered operation	Inventors or trained researchers may operate the prototype.	Non-specialists need simple steps, clear interpretation, error resistance, workflow fit, and meaningful decision outputs.	Conduct usability testing with representative users and environments; minimize manual steps and provide built-in checks.	[116,117,118,190,191,192,200]
Digital integration and traceability	Sensor signal may be reported without full data-system validation.	Secure hardware, software, calibration records, audit trails, privacy, cybersecurity, and data integrity.	Integrate chemistry, electronics, software, cloud systems, cybersecurity, dashboards, and controlled updates.	[110,111,112,113,114,115,116,117,144,145,146,147,148,149,150,151,152,153,154,155,156,157,158,159,160,161,162,163,164,165,166,167,168,169,170,213,214,215,216,231,232,233]
Regulation, market, and adoption	Publication value may dominate over regulatory pathway or business model.	Clear intended use, documentation, quality systems, certification, cost justification, service model, and user trust.	Engage standards early; evaluate total cost per useful decision, procurement, reimbursement, maintenance, and confirmatory testing needs.	[22,23,25,26,27,110,115,163,164,165,166,167,171,175,193,194,195,196,197,198,200,201,218,219,220,221,222,223,224,225,226,227,228,229,230,231,232,233,234,235,236,237,238,239,240,241,242,243]

## Data Availability

No new data were created or analyzed in this study. Data sharing is not applicable to this article.

## Abbreviations

The following abbreviations are used in this manuscript:

AI	Artificial intelligence
ATP	Adenosine triphosphate
CRISPR	Clustered regularly interspaced short palindromic repeats
DNA	Deoxyribonucleic acid
EHR	Electronic health record
FET	Field-effect transistor
GPS	Global positioning system
IoT	Internet of Things
ISFET	Ion-sensitive field-effect transistor
LAMP	Loop-mediated isothermal amplification
LFA	Lateral-flow assay
LFIA	Lateral-flow immunoassay
LOC	Lab-on-a-chip
LOD	Limit of detection
MEMS	Microelectromechanical systems
MIP	Molecularly imprinted polymer
ML	Machine learning
MOF	Metal–organic framework
NASBA	Nucleic acid sequence-based amplification
NIR	Near-infrared
OECT	Organic electrochemical transistor
PAD	Paper-based analytical device
PCR	Polymerase chain reaction
POC	Point-of-care
POCT	Point-of-care testing
QC	Quality control
QCM	Quartz crystal microbalance
RCA	Rolling circle amplification
RNA	Ribonucleic acid
RPA	Recombinase polymerase amplification
SERS	Surface-enhanced Raman scattering
SPR	Surface plasmon resonance
VOC	Volatile organic compound
WSN	Wireless sensor network
μPAD	Microfluidic paper-based analytical device
